# Sodium channels enable fast electrical signaling and regulate phagocytosis in the retinal pigment epithelium

**DOI:** 10.1186/s12915-019-0681-1

**Published:** 2019-08-15

**Authors:** Julia K. Johansson, Viivi I. Karema-Jokinen, Satu Hakanen, Antti Jylhä, Hannu Uusitalo, Maija Vihinen-Ranta, Heli Skottman, Teemu O. Ihalainen, Soile Nymark

**Affiliations:** 1 BioMediTech, Faculty of Medicine and Health Technology, Tampere University, Tampere, Finland; 2 Department of Biological and Environmental Science and Nanoscience Center, University of Jyväskylä, Jyväskylä, Finland; 3 Tays Eye Centre, Tampere University Hospital, Tampere, Finland

**Keywords:** RPE, Ion channels, Na_v_, Patch clamp, Phagocytosis, Retina, Photoreceptors

## Abstract

**Background:**

Voltage-gated sodium (Na_v_) channels have traditionally been considered a trademark of excitable cells. However, recent studies have shown the presence of Na_v_ channels in several non-excitable cells, such as astrocytes and macrophages, demonstrating that the roles of these channels are more diverse than was previously thought. Despite the earlier discoveries, the presence of Na_v_ channel-mediated currents in the cells of retinal pigment epithelium (RPE) has been dismissed as a cell culture artifact. We challenge this notion by investigating the presence and possible role of Na_v_ channels in RPE both ex vivo and in vitro.

**Results:**

Our work demonstrates that several subtypes of Na_v_ channels are found in human embryonic stem cell (hESC)-derived and mouse RPE, most prominently subtypes Na_v_1.4, Na_v_1.6, and Na_v_1.8. Whole cell patch clamp recordings from the hESC-derived RPE monolayers showed that the current was inhibited by TTX and QX-314 and was sensitive to the selective blockers of the main Na_v_ subtypes. Importantly, we show that the Na_v_ channels are involved in photoreceptor outer segment phagocytosis since blocking their activity significantly reduces the efficiency of particle internalization. Consistent with this role, our electron microscopy results and immunocytochemical analysis show that Na_v_1.4 and Na_v_1.8 accumulate on phagosomes and that pharmacological inhibition of Na_v_ channels as well as silencing the expression of Na_v_1.4 with shRNA impairs the phagocytosis process.

**Conclusions:**

Taken together, our study shows that Na_v_ channels are present in RPE, giving this tissue the capacity of fast electrical signaling. The channels are critical for the physiology of RPE with an important role in photoreceptor outer segment phagocytosis.

**Electronic supplementary material:**

The online version of this article (10.1186/s12915-019-0681-1) contains supplementary material, which is available to authorized users.

## Introduction

In the vertebrate eye, the retinal pigment epithelium (RPE) forms a barrier between the retina and the choroid [1–3]. Its cells are associated closely with photoreceptors: their apical sides surround the outer segments with long microvilli, and the basolateral sides are attached to Bruch’s membrane, an extracellular matrix separating the RPE from the choroid [3, 4]. The RPE has many functions that are vital to retinal maintenance and vision, such as maintaining the visual cycle, secreting important growth factors, delivering nutrients to the photoreceptors from the bloodstream while removing metabolic end products, and absorbing scattered light [1, 3]. Additionally, RPE maintains ionic homeostasis in the subretinal space [5] and sustains photoreceptor renewal by phagocytosing their shed outer segments [1, 6]. Phagocytosis is highly essential for vision, and it is under strict diurnal control, initiated at light onset for rods and typically at light offset for cones [7, 8]. This evolutionarily conserved molecular pathway is receptor mediated and precisely regulated; however, the exact signaling cascades are still not completely understood [9]. Recent studies imply the importance of specific ion channels in this process including the L-type calcium channels as well as calcium-dependent potassium and chloride channels [10–12].

Since the first single-cell recordings from RPE in 1988 [13], a large variety of different ion channels have been identified in them [5]. Among these are several voltage-gated calcium, potassium, and chloride channels. However, the identity of sodium conductive ion channels in RPE has remained elusive [5], even though the importance of sodium homeostasis to normal RPE function is acknowledged. Of the two main families of sodium channels, there is evidence of both epithelial Na^+^ channels and voltage-gated Na^+^ (Na_v_) channels in RPE [5, 14, 15, 18, 19]. However, electrophysiological data demonstrating their functionality is missing in mature RPE. More importantly, Na_v_ channels that are characteristic of excitable cells have to date only been detected from cultured RPE. This has resulted in the interpretation that their expression is due to neuroepithelial differentiation that can occur in culture [5, 20, 21].

Here, we shed light on this crucial issue by demonstrating the presence of Na_v_ channels both in cultured human embryonic stem cell (hESC)-derived RPE and freshly isolated mouse RPE. We show that Na_v_ channels co-regulate photoreceptor outer segment (POS) phagocytosis. Our hypothesis is supported by a recent demonstration of the involvement of Na_v_ channels in phagocytosis of mycobacteria by macrophages [22]. Our work provides evidence that Na_v_1.8 accumulates with the phagosomal particles. Na_v_1.4 also accumulates to phagosomes but displays localization to cell–cell junctions outside phagocytosis. Interestingly, selective Na_v_ channel blockers significantly reduced this phagosomal translocation. Moreover, the selective blockers combined with the universal Na_v_ blocker tetrodotoxin (TTX) reduced the total number of ingested POS particles by up to 41% while not affecting their binding. Reduction was also observed when the expression of Na_v_1.4 was silenced with short hairpin RNA (shRNA). More generally, our observations add to the growing body of evidence that Na_v_ channels play diverse roles in a variety of classically non-excitable cell types ranging from astrocytes and microglia to macrophages and cancer cells (for review, see [23]). Collectively, our results show that this epithelium is electrically more complex than was previously thought.

## Results

### Functional voltage-gated sodium channels are present in RPE derived from human embryonic stem cells

**Fig. 1:**
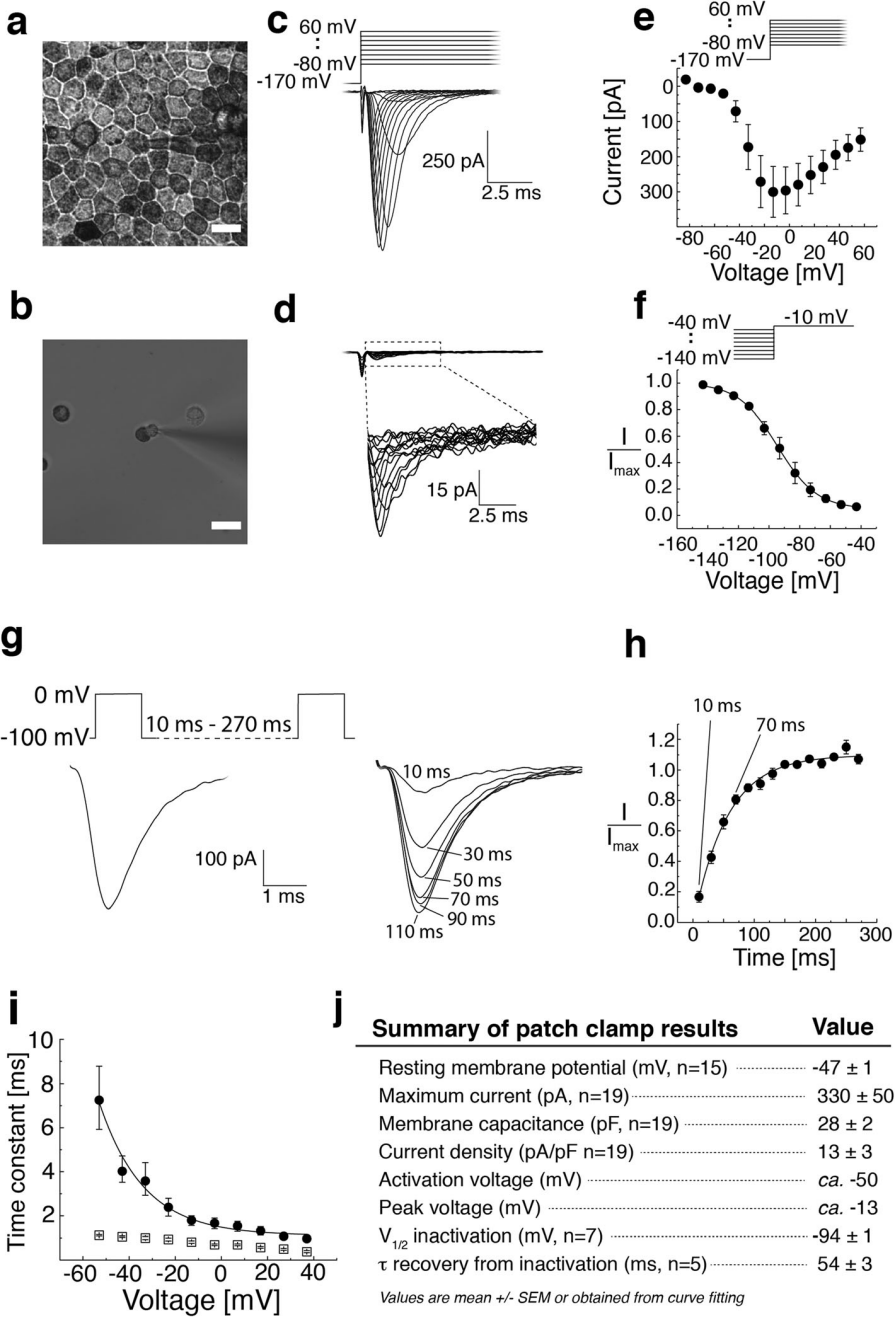
Patch clamp recordings of Na^+^ currents from hESC-derived RPE. **a**, **b** Brightfield light microscopy images of hESC-derived RPE cells. **a** Mature hESC-derived RPE grown on insert for 2 months showing strongly pigmented cells and characteristic epithelial morphology. **b** Mature hESC-derived RPE was dissociated yielding single cells with typical morphology showing pigmented apical and non-pigmented basal sides. Scale bars 10 μm. Whole-cell patch clamp recordings as responses to a series of depolarizing voltage pulses (− 80 to + 60 mV, 10 mV steps) after strong hyperpolarization (− 170 mV) either **c** from mature monolayer of hESC-derived RPE or **d** from single hESC-derived RPE cells. Patch clamp pipette is visible in the center of the **a** and **b** images. **e**–**i** Analysis of the monolayer recordings. **e** The average current–voltage relationship (*I* vs *V*_m_, mean ± SEM, *n* = 12). **f** Steady-state inactivation curve was analyzed by plotting the normalized peak current at − 10 mV test pulse against the prepulse voltage (− 140 to − 40 mV, 10 mV steps) and fitting the data with the Boltzmann equation. The best fit was obtained with *V*_1/2_ = − 94 ± 1 mV and *k* = 10 (*n* = 7). Data points indicate mean ± SEM. **g, h** The time dependency of recovery from inactivation. The second peak currents were normalized and plotted against the voltage pulse interval (10–270 ms). The best fit to an exponential function was obtained with τ = 54 ± 3 ms (*n* = 5) (individual datapoints for **h** available in Additional file 7: Table S2). **i** The activation (squares) and inactivation (circles) time constants were obtained from single exponential fits to the rising and decaying phases of the current responses shown in **c** and plotted against the command voltage (*n* = 7). **j** Summary of the patch clamp results

We used whole-cell recordings from mature hESC-derived RPE in K^+^ free intracellular solution to observe transient inward currents elicited by a series of depolarizing voltage pulses after strong hyperpolarization to − 170 mV (Fig. 1c, *n* = 19). These recordings were performed from an intact monolayer (Fig. 1a, results summarized in Fig. 1j) in the presence and absence of a gap-junction antagonist (18α-glycyrrhetinic acid). Resembling currents, but with only a fraction of the amplitude, were occasionally identified in cells from freshly dissociated mature hESC-derived RPE (Fig. 1b, d, *n* = 6), that is the conventional configuration for RPE patch clamp recordings. The current resembled the Na_v_ current characteristic of excitable cells: it had the typical current–voltage relationship (Fig. 1e) and showed fast activation and inactivation (Fig. 1i). The current was activated at about − 50 mV and peaked at about − 13 mV with a maximum amplitude of 330 ± 50 pA (mean ± SEM, *n* = 19). The average membrane capacitance was 28 ± 2 pF (*n* = 19), and the average current density was 13 ± 3 pA/pF (*n* = 19). The average resting membrane potential, measured in the presence of K^+^-based intracellular solution was − 47 ± 1 mV (mean ± SEM, *n* = 15). The inactivation time constant decayed exponentially with increasing command voltages, while the decay of the activation time constant was more shallow (Fig. 1i). The steady-state inactivation curve was determined by measuring the amplitude of a response to − 10 mV test pulse following a series of prepulses (from − 140 mV to − 40 mV at 10 mV intervals). The normalized current amplitude was plotted against the prepulse voltage and fitted with the Boltzmann equation

1 $$ I/{I}_{\mathrm{max}}(V)=1/\left\{1+\exp \left[\left(V-{V}_{1/2}\right)/k\right]\right\} $$

resulting in the half-inactivation voltage *V*_1/2_ = − 94 ± 1 mV (*n* = 7) (Fig. 1f). To investigate the time dependency of recovery from inactivation, we used a paired-pulse protocol (Fig. 1g). The current was recorded after a second depolarizing pulse given at increasing time intervals until it finally recovered to its full size. The second peak currents were subsequently normalized to the prepulse peak current and plotted against the time between the two voltage pulses (Fig. 1h). Our data was fitted with an exponential function, and the best fit yielded to *τ* = 54 ± 3 ms (*n* = 5).

**Fig. 2:**
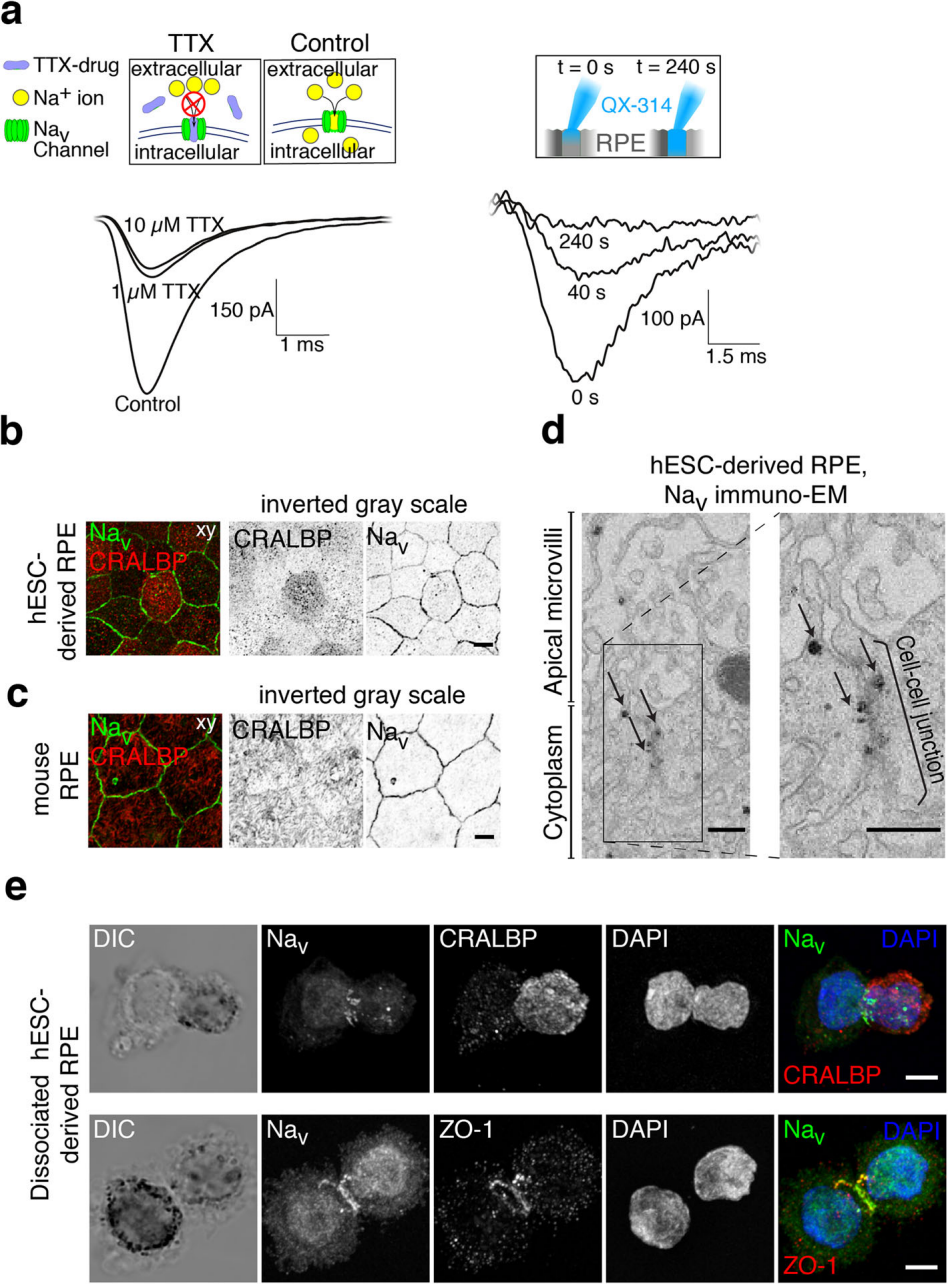
Blocker sensitivity and distribution of Na_v_ channels. Patch clamp recordings were performed on mature hESC-derived RPE monolayers. **a** Applying TTX extracellularly (either 1 μM or 10 μM) did not entirely block the current (left). The current was completely removed by intracellular QX-314 (2 mM) (right). Laser scanning confocal microscopy (LSCM) images on Na_v_ distribution in RPE cells. LSCM data inverted greyscale Z-maximum intensity projections of **b** hESC-derived and **c** mouse RPE stained against Na_v_ channels (green) and RPE marker CRALBP (red). Scale bars 10 μm. **d** Immunogold labeling and transmission electron microscopy images showing Na_v_ distribution at the apical membrane in the vicinity of the cell-cell junctions (black arrows). Scale bars 250 nm. **e** Dissociated hESC-derived RPE cells were let to adhere to poly-l-lysine coated coverslips for 30 min, fixed and immunolabeled against Na_v_ together with CRALBP (up) or tight junction marker ZO-1 (down). The Na_v_ label concentrated on the belt-like region in the middle of the cell, between the basal and apical sides. Scale bars 5 μm

The presence of Na_v_ currents was confirmed using the universal extracellular Na_v_ channel blocker TTX. By comparing the responses elicited with a voltage step from − 170 to − 10 mV, it was evident that addition of 1 μM TTX to the bath reduced the amplitude of the current to roughly one half of that recorded in the control extracellular solution (Fig. 2a, left). Thus, the recorded current was sensitive to TTX but required reasonably high concentrations. Furthermore, the sensitivity to TTX varied between the cells and in some cases even 10 μM TTX was not enough to block the current (Fig. 2a, left). The current was also sensitive to 2 mM QX-314, an intracellular Na_v_ channel blocker added to the internal solution of the patch pipette that typically removed the current rapidly after breaking into the whole-cell configuration (Fig. 2a, right).

### Voltage-gated sodium channels localize near cell–cell junctions in RPE

Our patch clamp data indicated that functional Na_v_ channels are present in the hESC-derived RPE. The cellular localization of the channels was investigated by performing immunofluorescence studies where the cellular retinaldehyde-binding protein (CRALBP), a marker for RPE cells [16, 17], was labeled together with the universal Na_v_ channel marker. These hESC-derived RPE samples were then imaged with a laser scanning confocal microscope (LSCM) by acquiring 3D image stacks (Fig. 2b), and the data were denoised by deconvolution. This showed that Na_v_ channels were present in fully differentiated RPE. Furthermore, the Na_v_ label concentrated primarily on the cellular borders with low expression elsewhere on the cell membrane while the CRALBP label was more uniformly localized to the apical side of the hESC-derived RPE (Fig. 2b).

Since the expression of Na_v_ channels in RPE has previously been thought to be induced in vitro by the cell culturing [18, 19] and since cells derived from ESCs might not fully replicate the pattern of ion channel expression in vivo [11, 20, 24–28], we wanted to confirm their presence by using freshly isolated and non-cultured mouse RPE (Fig. 2c). The same labeling showed highly similar distributions in mouse RPE as in hESC-derived RPE: the CRALBP label was cytoplasmic on the apical side of the cells while Na_v_ concentrated more on the cellular borders. Furthermore, the immunogold labeling for electron microscopy (immuno-EM) demonstrated the presence of Na_v_ channels in the cell–cell junctions (Fig. 2d) and our immunolabeling with the tight junction marker ZO-1 showed highly overlapping distributions, strongly suggesting the primary Na_v_ localization near the tight junctions (Additional file 1: Figure S1).

We investigated the mechanism underlying the previously reported lack of Na_v_ currents from acutely isolated RPE cells (Fig. 1d). The hESC-derived RPE cells were seeded on glass coverslips for 30 min and immunolabeled with the universal Na_v_ marker, CRALPB and ZO-1. Surprisingly, the Na_v_ label was primarily concentrated in the narrow region separating the apical and basolateral sides of the cell. Together with ZO-1, Na_v_ channels formed a clear ring-like structure between the apical and basal membranes following relaxation of junctional tension (Fig. 2e). Due to this junctional disruption, Na_v_ channels might not be accessible to pass ionic currents in acutely dissociated RPE cells.

### RPE cells express various voltage-gated sodium channel subtypes

**Fig. 3:**
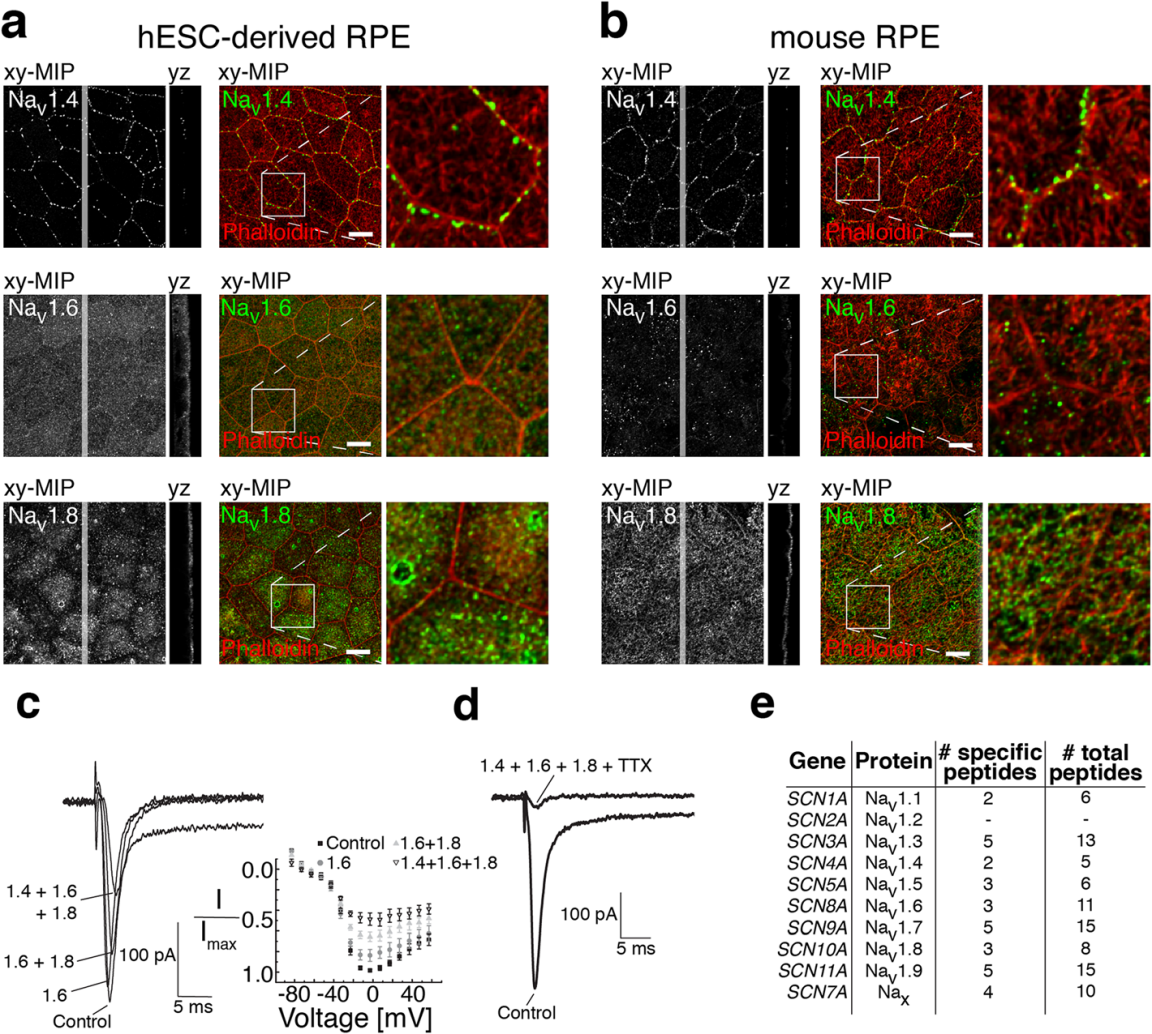
Immunolabeling of different Na_v_ subtypes in hESC-derived and mouse RPE, mass-spectrometry studies of Na_v_ expression, and patch clamp recordings with selective Na_v_ blockers. **a**, **b** The specific pattern of Na_v_ subtypes was studied by immunolabeling. Laser scanning confocal microscopy Z-maximum intensity projections (xy-MIP) and yz cross-sections of **a** mature hESC-derived or **b** mouse RPE. Na_v_ subtypes 1.4, 1.6, and 1.8 (green) were immunolabeled together with filamentous actin (phalloidin stain, red). Scale bars 10 μm. Right side panels show a higher magnification of the highlighted regions. Patch clamp recordings were performed on mature hESC-derived RPE using selective blockers for channel subtypes. **c** Na_v_ subtypes were sequentially blocked by extracellularly applied 4,9-AnhydroTTX (30 nM, Na_v_1.6 blocker), A-803467 (1 μM, Na_v_1.8 blocker) and μ-Conotoxin GIIB (600 nM, Na_v_1.4 blocker). The average normalized peak current–voltage relationship (*I*/*I*_max_ vs *V*_m_) was determined from all recordings (mean ± SEM, *n* = 7). **d** Applying the selective blockers in combination with TTX (10 μM) removed most of the Na_v_ currents (*n* = 11). **e** Mass spectrometry analysis of Na_v_ channel expression in hESC-derived RPE. Specific peptides were identified for all Na_v_ subtypes, excluding Na_v_1.2

Since ten different Na_v_ channel subtypes, Na_v_1.1–Na_v_1.9 and Na_x_, have been identified with drastically different expression profiles in diverse cell types, we wanted to investigate which specific channel subtypes are functionally expressed in the RPE cells. At the mRNA level, previous work has detected all of the Na_v_ channels in donated human RPE-choroid preparations, specifically Na_v_ subtypes 1.2–1.6 and Na_v_1.9 [29, 30]. We performed immunolabeling experiments with mouse and hESC-derived RPE using specific antibodies against channel subtypes Na_v_1.1–Na_v_1.9 (Fig. 3a, b, Additional file 2: Figure S2). Confocal microscopy showed that Na_v_1.4 localizes as beads-on-a-string to the cell–cell junctions (Fig. 3a, b). Na_v_1.8, on the other hand, localized overall to the apical side of the RPE cells (Fig. 3a, b). These data suggested that especially the Na_v_1.4 and Na_v_1.8 channels, which are usually expressed in skeletal muscle and dorsal root ganglia [31, 32], respectively, are also present in RPE cells. Na_v_1.6 the predominant channel of the adult central nervous system [33] showed a more homogenous labeling pattern in hESC-derived RPE and foci-like pattern in mouse RPE (Fig. 3a, b).

Subtypes Na_v_1.1, Na_v_1.3, Na_v_1.5, Na_v_1.7, and Na_v_1.9 were detected in cell–cell junctions and apical membrane but their labeling was more prominent after fixation with lower concentration of paraformaldehyde (Additional file 2: Figure S2). The subtype Na_v_1.2 was only weakly detected in both hESC-derived and mouse RPE. Additionally, we investigated the changes in channel subtype localization patterns during maturation of hESC-derived RPE (Additional file 3: Figure S3). The immunolabeling experiments indicated that the subtypes Na_v_1.4, Na_v_1.5, and Na_v_1.8 changed from homogeneous cellular distribution to more specific localization either to cell–cell junctions (Na_v_1.4) or to the apical side of the epithelium (Na_v_1.5 and Na_v_1.8) during the first 9 days of maturation.

To further verify the functional expression of the most prominent channel subtypes by electrophysiology, we repeated our patch clamp recordings using highly selective blockers for the channels Na_v_1.4, Na_v_1.6, and Na_v_1.8. The average current–voltage relationship (*I*–*V* curve) was determined from all these recordings (*n* = 7) (Fig. 3c). The current was sensitive to the combination of 30 nM 4,9-anhydro-TTX (Na_v_1.6 blocker), 1 μM A-803467 (Na_v_1.8 blocker), and 600 nM μ-conotoxin GIIB (Na_v_1.4 blocker), and the effect of inhibition was more potent with each added blocker thus confirming the expression and functionality of these channel subtypes in the hESC-derived RPE. However, the effect of inhibition was more significant when the blockers were combined with 10 μM TTX indicating the presence of Na_v_ subtypes additional to 1.4, 1.6, and 1.8 (*n* = 11) (Fig. 3d).

Finally, the channel subtype composition was verified by carrying out mass spectrometry (MS) analysis of gel bands obtained from hESC-derived RPE protein lysates that had been tested to show the main Na_v_ subtypes by Western blot (Additional file 4: Figure S4). Here, we followed the “two-peptide rule” [34], considering a hit positive if two or more specific peptides were identified. Intriguingly, all of the nine types, except subtype Na_v_1.2, were identified. This analysis thus further confirmed the expression of the three major subtypes (Na_v_1.4, Na_v_1.6, Na_v_1.8) in RPE and was also positive for the Na_x_ channel expression (Fig. 3e).

### Voltage-gated sodium channels Na_v_1.4 and Na_v_1.8 are involved in POS phagocytosis in RPE

Our previous experiments showed that several Na_v_ subtypes are present in both mouse and mature hESC-derived RPE. However, their physiological relevance remained unknown. Phagocytosis of POS is one of the major roles of RPE [3], and a plausible candidate function for the Na_v_ channels, as it requires rapid activation and high synchronization [35]. We therefore next investigated the potential importance of Na_v_ channels for POS phagocytosis.

**Fig. 4:**
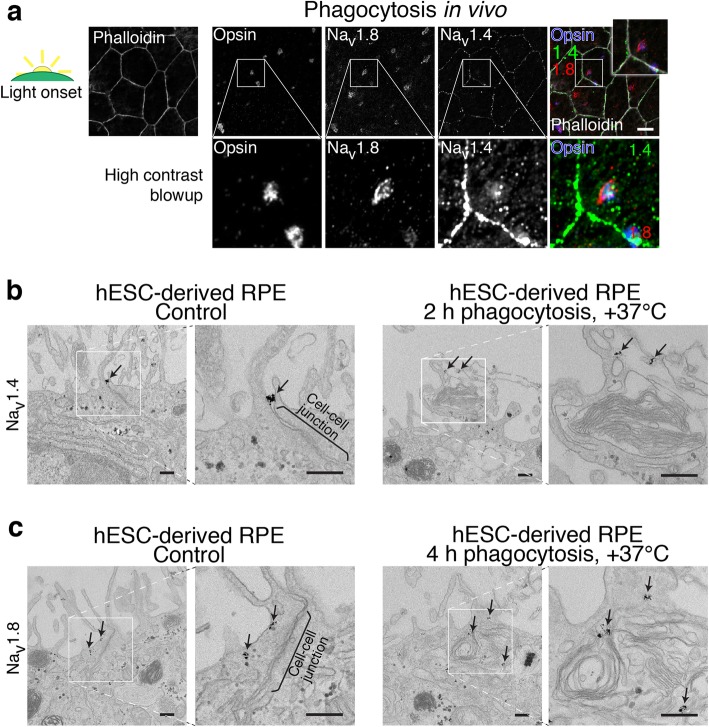
POS phagocytosis and the role of Na_v_1.4 and Na_v_1.8. **a** Phagocytosis was studied by dissecting mouse eyes at various time points during the circadian cycle. Filamentous actin was stained with phalloidin (gray in the merged image) to highlight epithelial cell–cell junctions. Laser scanning confocal microscopy Z-maximum intensity projections of mouse RPE prepared at light onset showed localization of opsin labeled POS particles (blue) and Na_v_1.4 (green) together with Na_v_1.8 (red). Lower panels show a high contrast blowup of highlighted regions. Scale bars 10 μm. To study phagocytosis in vitro, mature hESC-derived RPE were labeled with 1.4 nm nanogold-conjugated antibodies against **b** Na_v_1.4 and **c** Na_v_1.8 during phagocytosis of purified porcine POS particles and in control conditions. Without POS exposure, both channels showed localization near the cell-cell junctions (black arrows) but by incubating the monolayers with POS particles for **b** 2 h or **c** 4 h, the localization (black arrows) was also evident around the phagocytic cups and recently ingested phagosomes. Scale bars 250 nm

To study their role in the phagocytosis process, we performed immunolabeling experiments with mouse eyes that had been prepared at light onset near the diurnal peak of phagocytosis. The role of the channels in POS uptake was studied by comparing the immunolabeling of the three major subtypes (Na_v_1.4, Na_v_1.6 and Na_v_1.8) and opsin. Interestingly, at light onset, Na_v_1.4 and Na_v_1.8 localized to the bound POS particles (Fig. 4a). To confirm this redistribution of Na_v_ channels, we next performed immuno-EM experiments (Fig. 4b, c), where we labeled the subtypes with gold nanoparticles in hESC-derived RPE. When the cells had not been exposed to POS particles, the localization of both channel subtypes was junction adjacent. This labeling pattern was particularly evident for Na_v_1.4 (Fig. 4b) that formed clusters at the apical part of the cell–cell junctions. After 2 h or 4 h of phagocytosis, however, we could again observe the change in labeling distribution as the channels interacted directly with the phagocytic cups or recently ingested phagosomes (Fig. 4b, c).

**Fig. 5:**
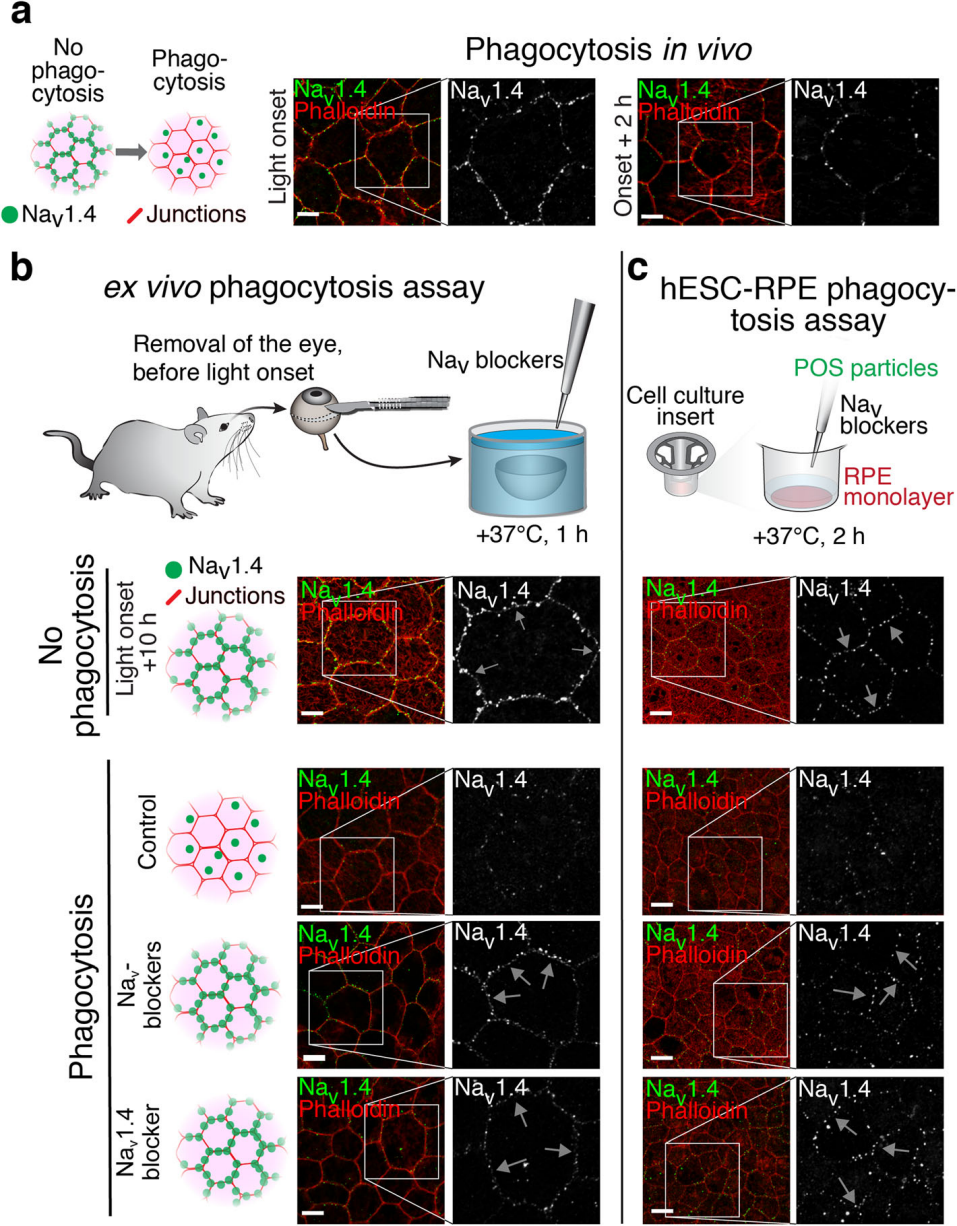
Redistribution of Na_v_1.4 during POS phagocytosis*.* The redistribution of Na_v_1.4 during phagocytosis and the effect of Na_v_ blockers to the process was studied in mouse and hESC-derived RPE. Filamentous actin was stained with phalloidin (red) to highlight epithelial cell-cell junctions. Laser scanning confocal microscopy Z-maximum intensity projections of **a** Na_v_1.4 localization in mouse RPE at light onset and 2 h after it showed strong reduction of the beads-on-a-string type labeling from cell–cell junctions. Different assays were used to investigate Na_v_1.4 distribution during phagocytosis and the effect of selective blockers for Na_v_1.4 (600 nM μ-Conotoxin GIIB) and Na_v_1.8 (1 μM A-803467) in combination with 10 μM TTX, or only of the selective blocker for Na_v_1.4. **b** The redistribution of Na_v_1.4 was studied ex vivo by incubating opened mouse eyecups in control solution or with the selective blockers. In both of the blocker samples, the redistribution was inhibited and the beads-on-a-string type labeling remained visible (white arrows) in the cell-cell junctions. **c** The hESC-derived RPE phagocytosis assay in vitro showed a highly similar redistribution of Na_v_1.4 and the blockers had the same effect as in the ex vivo mouse eyecup assay. Scale bars 10 μm

The redistribution of Na_v_ channels occurring during phagocytosis (Fig. 5a) was studied ex vivo with the channel blockers (Fig. 5b). For this purpose, we developed an assay where freshly opened mouse eyecups were incubated in physiological conditions with blocker solutions for 1 h starting at 15 min prior to light onset. The blocker for Na_v_1.4 as well as the combination of all Na_v_ blockers significantly prevented the disappearance of Na_v_1.4 from cell–cell junctions when compared to the control (Fig. 5b). The inhibition effect was similarly observed in hESC-derived RPE in vitro when the cells were incubated for 2 h with POS mixed with blocker solutions (Fig. 5c). We did not observe significant differences in the overall labeling pattern of Na_v_1.8 after the blocker incubation. Taken together, these experiments indicate the participation of Na_v_ channels in the phagocytic processes of RPE cells in vitro and in vivo.

### Na_v_1.4 knockdown and the inhibition of Na_v_ channels significantly reduces the number of ingested POS particles in hESC-derived RPE

**Fig. 6:**
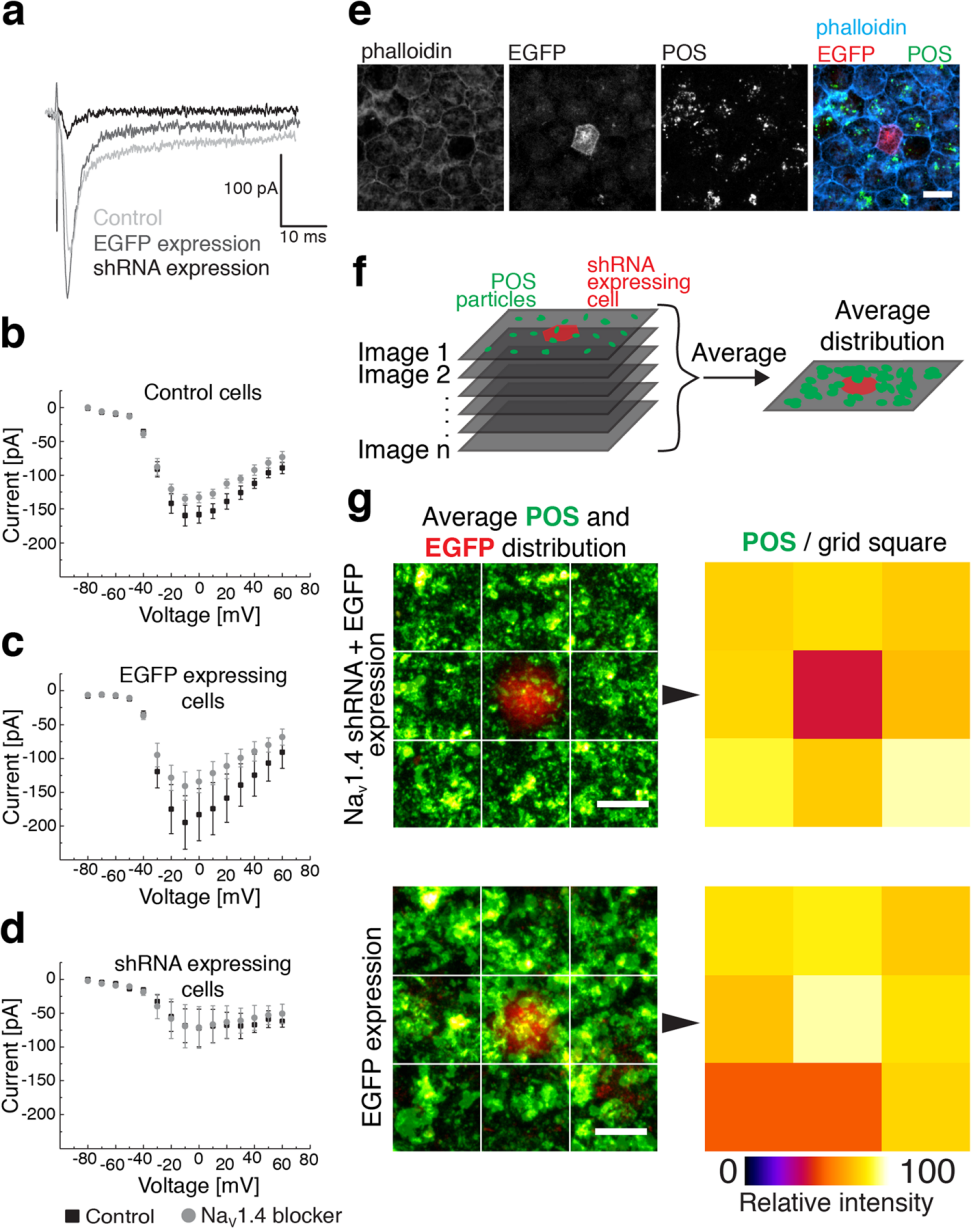
POS phagocytosis assay of shRNA Na_v_1.4 silenced hESC-derived RPE*.* Whole-cell patch clamp recordings were performed on mature hESC-derived RPE monolayers as responses to a series of depolarizing voltage pulses (− 80 to + 60 mV) after strong hyperpolarization **a** from control RPE cells, control vector cells (EGFP) and cells where Na_v_1.4 had been silenced with lentiviral vectors encoding shRNAs. The average current–voltage relationship (mean ± SEM,) was plotted for **b** Control hESC-derived RPE (*n* = 4), **c** EGFP expressing cells (*n* = 3), and **d** shRNA expressing cells (*n* = 3) (individual datapoints for **b**-**d** available in Additional file 8: Table S3). **e** The level of POS phagocytosis was analyzed with the EGFP expressing hESC-derived RPE cells. Filamentous actin was stained with phalloidin (blue) to highlight epithelial cell-cell junctions, EGFP (red) was used to identify the transduced cells and POS were labeled with opsin (green). **f** The average distribution of POS particles was analyzed from several images that had a single shRNA expressing cell placed in the middle. **g** The relative intensity of POS labeling in each square of the 3 × 3 grid was analyzed from Na_v_1.4 shRNA cells (*n* = 22 images) and control EGFP cells (*n* = 18 images). Scale bars 10 μm

Our LSCM and immuno-EM imaging of POS phagocytosis in RPE indicated a close interaction between Na_v_ channels and phagocytosed POS particles. Therefore, we hypothesized that reducing the Na_v_ channel activity could affect the rate of phagocytosis. After observing the dramatic change in the localization of Na_v_1.4, we decided to study its effect further by silencing the channel subtype expression by shRNAs (Fig. 6). Due to the challenges associated with passaging of hESC-derived RPE cells, such as loss of the cobblestone morphology and poor cell proliferation, we opted for the lentivirus shRNA constructs. The transduction of the RPE cells had to be conducted several days after the cell seeding yielding a monolayer with sparse distribution of single GFP-positive cells (Fig. 6e). Since it was not possible to confirm the knockdown efficiency in hESC-derived RPE, the constructs were first validated with ARPE-19 cells (Additional file 5: Figure S5). The cells were transduced with the shRNA constructs and collected for Western blot. Next, the knockdown effect of the verified construct was confirmed in hESC-derived RPE by conducting single cell patch clamp recordings and applying μ-conotoxin GIIB extracellularly (Fig. 6a-d). The cells expressing the target shRNA had both highly reduced Na_v_ currents and minimal reactivity to the blocker (Fig. 6d) when compared to EGFP-expressing (Fig. 6c) or wildtype hESC-derived RPE cells (Fig. 6b). Intriguingly, when the cells were used in the phagocytosis assay (Fig. 6e), the silencing of Na_v_1.4 caused a drastic reduction in the total number of POS particles found in individual GFP-positive cells on the monolayer (Fig. 6f, g).

**Fig. 7:**
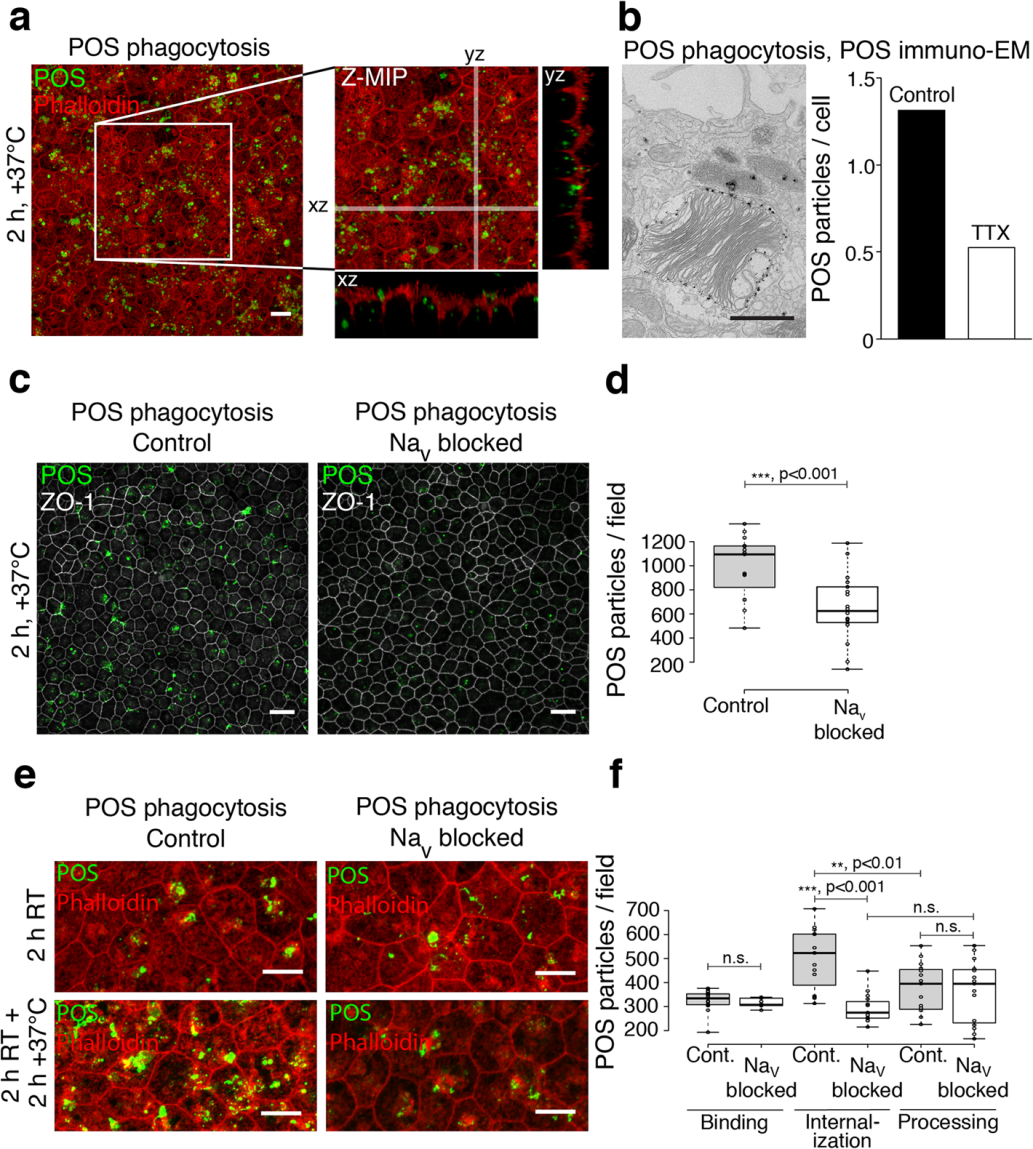
POS phagocytosis assay of hESC-derived RPE with selective Na_v_ blockers. POS phagocytosis assays were performed on mature hESC-derived RPE by incubating the monolayers with purified porcine POS particles with or without Na_v_ blockers (600 nM μ-Conotoxin GIIB, 1 μM A-803467 and 10 μM TTX). **a** Laser scanning confocal microscopy (LSCM) Z-maximum intensity projections (Z-MIP) and yz and xz cross-sections of the RPE and POS particles (green) with filamentous actin staining (red) after 2 h of phagocytic challenge. Scale bar 10 μm. **b** Quantification of POS particles from TEM images with immunogold labeled opsin showed a 60% decrease of POS particles/cell in the presence of the blockers. Scale bar 250 nm. **c** LSCM Z-MIP images of ZO-1 (gray) together with opsin (green). Scale bars 20 μm. **d** Quantification of control (*n* = 15) and Na_v_ blocker samples (*n* = 18) during phagocytosis (2 h +37 °C) showed a 34% reduction in the total number of POS particles in the presence of Na_v_ blockers. **e**, **f** Na_v_ channel role in POS binding, internalization and further processing. **e** LSCM Z-MIP images of phalloidin (red) together with opsin (green) during POS binding and internalization. Scale bars 10 μm. **f** Quantification of the binding phase (2 h RT, Control *n* = 15, Na_v_ blockers *n* = 10) showed no significant reduction in POS numbers due to Na_v_ channel blockers, but a 41% decrease in the internalization phase (2 h RT + 2 h at +37 °C, Control *n* = 15, Na_v_ blocker *n* = 15). In control cells, the POS numbers decreased in the processing phase, but not in the presence of Na_v_ channel inhibitors (2 h RT + 5 h at +37 °C, 25% decrease, Control *n* = 15, Na_v_ blocker *n* = 15). Center lines show the medians; box limits indicate the 25th and 75th percentiles as determined by R software and whiskers extend to minimum and maximum values

To then study the effect of all Na_v_ channels on a larger population of cells, we performed the in vitro phagocytosis assay (Fig. 7a) in the presence of Na_v_1.4 and 1.8 blockers and TTX. The effect was first quantified by counting the number of particles from the immuno-EM images that had been tagged with gold nanoparticle labeled opsin (Fig. 7b). This revealed a drastic reduction in the total number of bound and internalized POS particles. To better analyze the effect, the assay was carried out by imaging large fields of immunolabeled opsin and ZO-1 and by comparing the number of POS particles in Na_v_ blocker and control conditions after 2 h at +37 °C (Fig. 7c). The results showed that the blocker combination caused a 34% (*n* = 18) reduction in the total number of POS particles labeled with opsin (Fig. 7d).

Next, we wanted to independently quantify the binding and internalization stages of the phagocytosis pathway. The binding was investigated by incubating the POS-supplemented hESC-derived RPE cells at room temperature (RT) [35, 36] with 5% CO_2_. Most of the unbound POS were then removed by gentle washing, and the monolayers were placed in the incubator for either 2 h or 5 h to investigate the internalization stage of phagocytosis. This was carried out also with the combination of Na_v_ blockers and the total number of POS particles was compared to control cells at each timepoint (Fig. 7e, f). The results showed no statistically significant changes (*p* = 0.1) in the number of POS particles due to the Na_v_ inhibition in the particle binding phase (2 h RT, Control *n* = 15, Na_v_ blockers *n* = 10). However, Na_v_ blocking caused a 41% reduction of POS particle numbers in the internalization phase (2 h RT + 2 h at +37 °C, Control *n* = 15, Na_v_ blocker *n* = 15) (*p* < 0.001). In control cells, the internalized particles were degraded, which was detected as a 25% reduction in particle numbers in the processing phase (2 h RT + 5 h at +37 °C, *n* = 15, *p* < 0.01) (Fig. 7f). Na_v_ blocking substantially reduced further processing of POS particles in RPE cells as there were no significant changes in particle numbers between internalization and processing phases (*n* = 15, *p* = 0.16). Together, our data indicate that functional Na_v_ channels directly interact with phagosomes (Fig. 4) and that they are involved in the POS particle internalization and further processing (Fig. 7e, f).

## Discussion

Recent studies show revolution in our understanding of the roles that Na_v_ channels have in cellular functions; no longer are these proteins considered important only in “classically” electrically excitable tissues. Here, we provide the first evidence, to our knowledge, that Na_v_ channels are expressed in the epithelial cells (Figs. 1, 2, and 3, Additional file 1: Figure S1, Additional file 2: Figure S2) and that their activity co-regulates phagocytosis in RPE. Our observations of Na_v_ channels and Na_v_-mediated currents in intact RPE preparations (mature hESC-derived RPE monolayers and freshly isolated mouse RPE) demonstrate that previous observations of Na_v_-mediated currents in cultured RPE cells are not preparation-dependent artifacts [18, 19]. Rather, the absence of Na_v_-mediated currents in acutely isolated RPE cells (Fig. 1d) likely results from the destruction of tight junction complexes during dissociation (Fig. 2e). Internalization of Na_v_ channels, of course, would result in diminution or absence of membrane currents mediated by these channels as observed by us (Fig. 1d) and others [18, 19]. Reductions in Na_v_ currents have also been reported in dorsal root ganglion neurons following peripheral axotomy [37, 38] but this effect was found to be reversible by exposure to growth factors [37, 38]. The observation of Na_v_ currents in recordings from hESC-derived RPE monolayers, we believe, is strong evidence that cells in RPE with intact tight junctions usually express functional Na_v_ channels in their plasma membranes.

The properties of Na_v_-mediated currents in hESC-derived RPE cells are consistent with the earlier recordings from other non-neuronal cells [23]. Furthermore, non-excitable cells have been shown to display varying sensitivity to TTX based on their Na_v_ subtype composition [23], and our recordings from RPE align with this observation. The high TTX concentration needed for a full inhibition of the current is supported by the finding that RPE cells were labeled strongly by an anti-Na_v_1.8 antibody; Na_v_1.8 is the least sensitive of Na_v_ channels to TTX [39]. Our pharmacological analysis of Na_v_-mediated currents using Na_v_ subtype-specific blockers, immunofluorescence microscopy and MS-based analysis (Fig. 3, Additional file 2: Figure S2) indicated that Na_v_1.1 as well as Na_v_1.3–Na_v_1.9 subtypes are present in the RPE together with the sodium concentration sensitive Na_x_ channel [40]. This data was further supported by the Na_v_ current characteristics: the relatively slow recovery from inactivation [41, 42] (Fig. 1h) and the large variation visible in the early phase of the recovery, indicate the presence of several different Na_v_ subtypes in the RPE. Compared to neurons, other non-excitable cells, such as non-process bearing astrocytes and supporting cells of the porcine vomeronasal organ [43–45], have been shown to exhibit more negative steady-state half-inactivation voltages, and similar *V*_1/2_ values were found for hESC-derived RPE (Fig. 1f). Accordingly, and similarly to astrocytes [46], we never observed spontaneous action potentials in these cells. Earlier recordings from RPE have shown *V*_1/2_ values much closer to neurons; however, these results were obtained after only a short period in culture [15, 19]. As our data demonstrates that the Na_v_ channel localization and subtype composition is dynamically regulated during development (Additional file 3: Figure S3), we believe that RPE maturation stage including the junctional localization of the channels could greatly influence their electrophysiological properties. This is supported by the previous recordings that identified Na_v_ channels in astrocytes in hippocampal slices [46].

For the MS-based identification of Na_v_ proteins, we chose to perform the analysis from gel bands [47–49] due to the high sensitivity and possibility for absolute protein identification [48] provided by this approach. However, it was challenging to purify the Na_v_ channel proteins from the RPE cells, most probably due to their several transmembrane domains and high hydrophobicity [50]. Therefore, the number of detected Na_v_ channel-specific peptides does not necessarily directly correlate to their relative abundance. Na_v_1.4 and Na_v_1.8 of the identified Na_v_ subtypes showed the strongest staining in the immunohistochemical analysis and carried over one third of the total current in patch clamp recordings. Therefore, we focused on these two channel subtypes while investigating the physiological roles of Na_v_ channels. It is noteworthy, however, that the subtypes Na_v_1.1, Na_v_1.3, Na_v_1.5, and Na_v_1.7 only showed strong junctional labeling after fixation with 1% PFA. Our antibodies labeling these subtypes are directed against different residues of the same intracellular loop between the domains III and IV. Importantly, this region might be sequestered in the dense cell–cell junctions thus hampering their detection through conventional immunocytochemistry [51]. On the other hand, cells derived from ESCs can bear differences compared to native cells regarding subtype composition. Earlier studies have demonstrated high similarity but also certain deviations in terms of ion channel distribution and biophysical characteristics as well as channel pharmacology and transcriptional profile [11, 20, 24–28].

Previous studies have shown that in macrophages, Na_v_ channels have important roles in phagocytosis [22, 52–54]. We observed an accumulation of Na_v_1.4 and Na_v_1.8 towards POS particles during phagocytosis. The translocation of Nav1.4 from tight junctions was still evident 2 h after light onset (Fig. 5a), near the peak expression level of a phagocyte cell surface tyrosine kinase receptor MerTK [55]. The involvement of Na_v_1.4 in phagocytosis was supported by the fact that its silencing decreased the amount of POS particles (Fig. 6). Furthermore, following Na_v_ blocker incubation, we observed a decrease in its translocation (Fig. 5) with a concurrent reduction in the number of POS particles (Fig. 7). Na_v_ channel redistribution has been observed in demyelinated axonal membrane [56] suggesting that these ion channels can display dynamic regulation of distribution and have important implications in various pathologies.

Participation of Na_v_ channel activity to POS phagocytosis was further indicated by their direct association with both the forming phagocytic cups and ingested phagosomes (Fig. 4). Although inhibition of Na_v_ channels did not abolish phagocytosis, the observed 41% attenuation (Fig. 7) was similar to the previously reported effect of TTX in microglia [57]. Our assays suggest that the Na_v_ channels are involved in the engulfment and further processing of the phagosomes, since inhibiting Na_v_ activity did not impair the binding of POS particles (Fig. 7). Interestingly, changes in intracellular free calcium concentration regulate phagocytosis in RPE [58] and, more specifically, particle engulfment in other phagocytes [59]. Na_v_ mediated sodium influx could result in increased calcium concentration via reversed functioning of sodium–calcium exchangers expressed in the RPE apical membrane [60, 61] thus affecting phagocytosis. Alternatively, Na_v_ channels could regulate endosomal acidification by providing a sodium efflux pathway to enhance the entry of protons, similarly to macrophages [22, 53]. In these cells, phagocytosis has been associated with membrane potential hyperpolarization due to the activation of a Ca^2+^-dependent K^+^ conductance [62, 63]. Such change in potential could relieve the Na_v_ channel inactivation in the early phase of phagocytosis, and the channels could subsequently be activated in the phagosomes and endosomes with membrane potential in the Na_v_ activation range [64–66]. Furthermore, the channels could be involved directly in the circadian control of the pathway, as has been recently shown for other ion channels [10].

The fact that RPE expresses such a versatile array of Na_v_ channels suggests that besides phagocytosis, these channels also have other roles in the physiology of the RPE. Overall, sodium homeostasis is critical to epithelial transport mechanisms, and our observation of Na_v_ channels, including the non-voltage-gated Na_x_ channel, brings a new piece in the ongoing identification of the sodium conducting proteins in RPE. In excitable cells, Na_v_ and Ca^2+^ channels form local signaling complexes that are essential for various intracellular processes [67]. Similar roles for these channels could be possible in RPE as well. Moreover, epithelial cells, including RPE, show strong calcium waves in response to mechanical stimulation [68–70], and it is likely that Na_v_ channels are involved in the process. It is well established that the Ca^2+^ binding protein calmodulin (Cam) interacts directly with the C-terminal domain of Na_v_ [71], and it was recently shown that the Ca^2+^-free form of Cam, ApoCam, enhances the Na_v_ channel opening by several-fold [72]. Thus, Na^+^- and Ca^2+^-dependent signaling pathways can interact in epithelia as has been reported in the case of astrocytes [73]. Lastly, it has been suggested that retinal Müller cells that display similar low Na_v_ channel densities could be activated by the adjacent neurons and it is possible that RPE cells could also serve as voltage sensors reacting to signals arising from photoreceptors [44, 74].

## Conclusion

The results of this study demonstrate that functional Na_v_ channels are present in mouse and hESC-derived RPE cells with intact tight junctions. Specifically, we confirm the presence of Na_v_1.1 as well as Na_v_1.3–Na_v_1.9 subtypes and the sodium concentration sensitive Na_x_ channel, showing that their expression is not due to specific culturing conditions. Our data shows that the most prominent subtypes Na_v_1.4 and Na_v_1.8 are involved in photoreceptor outer segment renewal by directly interacting with phagosomes. Inhibiting the activity of these channels by either pharmacological blockers or shRNA-mediated silencing impairs the phagocytosis process, particularly at the engulfment or further processing stages. Collectively, we demonstrate that Na_v_ channels yield RPE cells the capacity for fast voltage sensitivity and that the channels are a vital part of its physiology.

## Methods

### Antibodies and reagents

Catalog and batch numbers as well as other information for the chemicals and antibodies used in this study can be found from the Additional file 6: Table S1.

### Cell culturing

Human ESC lines Regea08/023 and Regea08/017 were cultured as previously described [11, 75]. Briefly, the hESC-derived RPE were spontaneously differentiated in floating cell clusters. The pigmented areas were isolated manually and the cells were dissociated with Tryple Select (1X, Thermo Fisher Scientific) and filtered through cell strainer (BD Biosciences, NJ, USA). The isolated cells were then seeded on collagen IV-coated (human placenta, 5 μg/cm^2^; Sigma-Aldrich, MO, USA) 24-well plates (NUNC, Thermo Fisher Scientific, Tokyo, Japan) for enrichment. Subsequently, the pigmented cells were replated for maturation on culture inserts (Millicell Hanging Cell Culture Insert, polyethylene terephthalate, 1.0 μm pore size, EMD Millipore, MA, USA) coated either with Collagen IV (10 μg/cm^2^) or with collagen IV and laminin (1.8 μg/cm2, LN521, Biolamina, Sweden). The cells were cultured at +37 °C in 5% CO_2_ in culture medium consisting of Knock-Out Dulbecco’s modified Eagle’s medium (KO-DMEM), 15% Knock-Out serum replacement (KO-SR), 2 mM GlutaMax, 0.1 mM 2-mercaptoethanol (all from Life Technologies, Carlsbad, CA), 1% Minimum Essential Medium nonessential amino acids, and 50 U/mL penicillin/streptomycin (from Cambrex BioScience, Walkersville, MD, USA). The culture medium was replenished three times a week. Mature monolayers typically showed transepithelial resistance values (TER) of over 200 Ω cm^2^.

### Generation of Na_v_1.4 shRNA cultures

ARPE-19 cells (ATCC, USA) were maintained in DMEM/F12 medium containing 10% FBS, 1% GlutaMAX and 1% penicillin/streptomycin at 37 °C with 5% CO_2_. The medium was changed 3 times a week. The confluent cells were dissociated with trypsin-EDTA (25200–056, Thermo Fisher Scientific) and transfected the following day with shRNA expression vectors containing the reporter vector pLKO.1-CMV-tGFP or pLKO.1-puro-CMV-TurboGFP (Sigma-Aldrich). The expression of shRNA was then investigated by Western blot analysis for Na_v_1.4 for clones TRCN0000416043, TRCN0000425151, and TRCN000044419. This was carried out by comparing the labeling intensity against β-actin that was used as the loading control (*n* = 3). The normalization was carried out by subtracting the background intensity (${I}_{Nav}^{Bgrnd},{I}_{Actin}^{Bgrnd}$) from the intensity of Na_v_ and β-actin bands (${I}_{Nav}^{Band},{I}_{Actin}^{Band}$). The bands were then normalized to the maximum intensity (${I}_{Nav}^{Max},{I}_{Actin}^{Max}$), yielding normalized Western blot band intensities between values 0 and 1 as follows:

2 $$ {I}_{Nav}^{Norm}=\frac{I_{Nav}^{Band}-{I}_{Nav}^{Bgrnd}}{I_{Nav}^{Max}-{I}_{Nav}^{Bgrnd}}\ \mathrm{and}\ {I}_{Actin}^{Norm}=\frac{I_{Actin}^{Band}-{I}_{Actin}^{Bgrnd}}{I_{Actin}^{Max}-{I}_{Actin}^{Bgrnd}}. $$

These normalized intensities were then used to calculate the relative knockdown as

3 $$ {I}_{knockdown}=\frac{I_{nav}^{Norm}}{I_{Actin}^{Norm}}. $$

After Western blot analysis, 2 μl of the verified clone TRCN000044419 (8.1 × 10^6^ TU/ml) and 8 mg/ml polybrene were added on hESC-derived RPE cells grown on insert. Transduction was made 5–23 days after the cell seeding and virus particles were incubated for 1 day before changing medium. The silencing of Na_v_1.4 current was verified by patch clamp from maturated, 8–10 weeks old, hESC-derived RPE cells.

### Sample preparation

For monolayer patch clamp recordings and immunolabeling, the membrane of the culture insert was removed from the insert holder and cut into smaller pieces. The cells were rinsed three times either with PBS (for immunolabeling) or with Ames’ solution (for patch clamp recordings). For the experiments on dissociated cells, the hESC-derived RPE monolayers were treated with TrypLE Select for 10 min in +37 °C, gently mechanically triturated with a pipette and centrifuged for 5 min at 1000 rpm. Dissociated cells were resuspended in culture medium, seeded on glass coverslips coated with poly-l-lysine (Sigma-Aldrich) and allowed to settle down for 10 min for patch clamp recordings and 30 min for immunolabeling.

Mouse RPE was prepared for immunolabeling as follows. C57BL/6 mice were euthanized by CO_2_ inhalation and cervical dislocation. The eyes were enucleated and bisected along the equator, and the eyecups were sectioned in Ames’ solution buffered with 10 mM HEPES and supplemented with 10 mM NaCl, pH was adjusted to 7.4 with NaOH (Sigma-Aldrich). The retina was gently removed from the eyecup leaving the RPE firmly attached to the eyecup preparation.

### Patch clamp recordings

Ionic currents were recorded from mature hESC-derived RPE monolayers or freshly dissociated cells using the standard patch clamp technique in whole-cell configuration. Patch pipettes (resistance 5–6 MΩ) were filled with an internal solution containing (in mM) 83 CsCH_3_SO_3_, 25 CsCl, 10 TEA-Cl, 5.5 EGTA, 0.5 CaCl_2_, 4 ATP-Mg, 0.1 GTP-Na, 10 HEPES, and 5 NaCl; pH was adjusted to ~ 7.2 with CsOH and osmolarity was ~ 290 mOsm (Gonotec, Osmomat 030, Labo Line Oy, Helsinki, Finland). For the recordings with K^+^-based internal solution, CsCl was replaced with KCl and CsCH_3_SO_3_ was replaced with K-gluconate. In some experiments, the internal solution also contained 2 mM QX-314-Cl (from Sigma-Aldrich). During all recordings, the tissue was perfused at 2.5 ml min^−1^ with Ames’ solution (Sigma-Aldrich) buffered with 10 mM HEPES and supplemented with 10 mM NaCl and 5 mM TEA-Cl. The pH was adjusted to 7.4 with NaOH and the osmolarity set to ~ 305 mOsm. The bath solution contained 10 nM-10 μM TTX citrate (from Tocris Bioscience) when the effect of TTX on the recorded currents was investigated, and 30 μM 18α-glycyrrhetinic acid (from Sigma-Aldrich) when the effect of gap junctional coupling was tested. For the channel subtype recordings, the bath solution was supplemented with 30 nM 4,9-AnhydroTTX, 1 μM A-803467, or 600 nM μ-Conotoxin GIIB. All recordings were made in voltage clamp mode with pClamp 10.2 software using the Axopatch 200B patch clamp amplifier connected to an acquisition computer via AD/DA Digidata 1440 (Molecular Devices, USA). The access resistance was below 30 MΩ and the membrane resistance above 150 MΩ. Series resistance was 15–30 MΩ and was not compensated. Holding potentials were corrected for a 3 mV liquid junction potential during the data analysis. All recordings were performed at room temperature.

### Immunolabeling

Prior to immunolabeling, samples were washed three times with PBS and fixed for 15 min with 4% paraformaldehyde or 10 min with 1% paraformaldehyde (pH 7.4; Sigma-Aldrich). After repeated washes with PBS, samples were permeabilized by incubating in 0.1% Triton X-100 in PBS (Sigma-Aldrich) for 15 min and subsequently blocked with 3% BSA (BSA; Sigma-Aldrich) for 1 h. All immunolabeling incubations were done at room temperature.

Primary antibodies against the following proteins were used in this study: cellular retinaldehyde-binding protein (CRALBP) 1:400 (ab15051, Abcam), Na_v_1.1 1:200 (ASC-001, Alomone labs), Na_v_1.2 1:200 (ab99044, Abcam), Na_v_1.3 1:200 (ASC-004, Alomone labs), Na_v_1.4 1:200 (ASC-020, Alomone labs), Na_v_1.5 1:200 (AGP-008, Alomone labs), Na_v_1.6 1:200 (ASC-009, Alomone labs), Na_v_1.7 1:200 (ASC-008, Alomone labs), Na_v_1.8 1:200 (AGP-029, Alomone labs), Na_v_1.9 1:200 (AGP-030, Alomone labs), Pan Na_v_ (Na_v_) 1:200 (ASC-003, Alomone labs), and Zonula occludens-1 (ZO-1) 1:50 (33-9100, Life Technologies). All primary antibodies were diluted in 3% BSA in PBS and incubated for 1 h.

The incubation with primary antibodies was followed by three PBS washes and 1 h incubation with secondary antibodies; goat anti-rabbit Alexa Fluor 568 (A-11011), donkey anti-rabbit Alexa Fluor 488 (A-21206), donkey anti-mouse Alexa Fluor 568 (A10037), donkey anti-mouse Alexa Fluor 488 (A-21202), goat anti-guinea pig Alexa Fluor 568 (A-11075)**,** goat anti-mouse Alexa Fluor 488 (A-11029), donkey anti-rabbit Alexa 647 (A-31573), donkey anti-mouse Alexa 647 (A-21236), goat anti-guinea pig Alexa Fluor 647 (A-21450) and goat anti-mouse Alexa Fluor 405 (A-31553) (all from Molecular Probes, Thermo Fisher Scientific) diluted 1:200 in 3% BSA in PBS. Actin was visualized using either a direct phalloidin Alexa Fluor 647 conjugate 1:50 (A22287, Thermo Fisher Scientific), Atto-633 1:50 (68825, Sigma-Aldrich) or tetramethylrhodamine B conjugate 1:400 (P1951, Sigma-Aldrich) and the nuclei were stained with 4′, 6′-diamidino-2-phenylidole (DAPI) included in the ProLong Gold antifade mounting medium (P36935, Thermo Fisher Scientific).

### Pre-embedding immunogold labeling

The hESC-derived RPE monolayers were washed three times with phosphate-buffered saline (PBS) and then fixed for 2 h at RT in periodate-lysine-paraformaldehyde (PLP) fixative. Fixed cells were prepared for pre-embedding EM as described previously [76, 77]. Cells were treated with 0.01% saponin and 0.1% BSA in 0.1 M phosphate buffer, pH 7.4 (Buffer A) before adding the primary antibodies diluted in Buffer A. The concentration of all primary antibodies was doubled for the experiment compared to immunolabeling. After 1 h incubation at RT and washes with Buffer A, 1.4 nm nanogold-conjugated polyclonal Fab’ fragment of goat anti-rabbit IgG or of goat anti-mouse IgG (Nanoprobes.com, Yaphank, NY, USA) diluted to 1:50 in Buffer A was applied for 1 h, followed by washes with Buffer A and 0.1 M phosphate buffer (pH 7.4). Cells were post-fixed with 1% glutaraldehyde in phosphate buffer for 10 min at RT, quenched with 50 mM NH_4_Cl in phosphate buffer for 5 min at RT, and then washed with phosphate buffer and water.

The samples were treated in dark with HQ-silver (Nanoprobes.com) for 5 min followed by washes with water and gold toning (2% sodium acetate 3 × 5 min at RT, 0.05% gold chloride 10 min at +4 °C, 0.3% sodium thiosulphate 2 × 10 min at +4 °C). After washes with water, the cells were reduced in 1% osmium tetroxide in 0.1 M phosphate buffer for 1 h at +4 °C and dehydrated with graded series of ethanol (70%, 96%, 100%), then stained with 2% uranyl acetate. Finally, the monolayers were embedded in Epon (TAAB Embedding resin, medium, TAAB Laboratories Equipment Ltd., Berks, UK) and after polymerization, sections perpendicular to the membrane were cut with an ultramicrotome (Leica ultracut UCT ultramicrotome, Leica Mikrosysteme GmbH, Austria). The thin sections (200 nm) were placed on carbon-coated single-slot grids and were imaged with JEOL JEM-1400 transmission electron microscope (JEOL Ltd., Tokyo, Japan) equipped with bottom-mounted Quemesa CCD camera (4008 × 2664 pixels). High voltage of 80 kV was used for imaging.

### Western blotting

The hESC-derived RPE and ARPE-19 protein lysates were obtained by incubating 1 × 10^6^ cell pellets in RIPA buffer supplemented with Halt protease inhibitor cocktail (87786, Thermo Fisher Scientific) for 30 min at +4 °C on constant agitation. The lysate was then centrifuged at +4 °C for 20 min at 12,000×*g*, mixed with Novex sample buffer (NP0007, Thermo Fisher Scientific) and heated at +70 °C for 10 min. The protein lysates were then loaded onto 3–8% NuPage gel (EA0375, Thermo Fisher Scientific) or Bolt™ 4–12% Bis-Tris Plus Gels (NW04120, Thermo Fisher Scientific), fractionated by SDS-PAGE and then either processed for MS analysis or transferred to nitrocellulose membrane via Trans Blot Turbo Transfer system according to the manufacturer’s protocols (BioRad).

The resulting blot was blocked with 3% BSA in PBS + 0.1% Tween-20 5 h at RT and then labeled overnight at +4 °C with the primary antibodies against various Na_v_ subtypes diluted in blocking solution. The following antibodies were labeled with this protocol: Na_v_1.4 1:500 (PA5-36989, Thermo Fisher Scientific), Na_v_1.5 1:500 (AGP-008, Alomone labs), Na_v_1.6 1:1000 (ASC-009, Alomone labs), and β-actin 1:2000 (ab6276, Abcam). The membranes were subsequently washed three times for 15 min with PBS + 0.1% Tween-20 and incubated with a 1:20,000 dilution of horseradish peroxidase-conjugated goat anti-rabbit IgG (ab6721, Abcam), goat anti-guinea pig IgG (ab6908, Abcam), or anti-mouse IgG (A-21236, Thermo Fisher Scientific) antibodies for 1 h at RT. For Na_v_1.8 1:5000 (ASC-016, Alomone labs), the protocol was modified as follows: blocking was overnight at +4 °C, primary antibody labeling was for 1 h at RT, washing was three times for 10 min with PBS + 0.01% Tween-20 and the secondary antibody was incubated with a 1:3000 dilution for 1 h at RT. After subsequent washes, the membranes were developed with the WesternBright ECL system (K-12045-D20, Advansta) and imaged with ChemiDoc XRS+.

### Sample preparation of mass spectrometry

The SDS-page gels were labeled overnight at RT with coomassie blue dye to identify the bands. Protein bands ranging from 200 to 260 kDA were excised from the gel and destained by submerging the samples in acetonitrile (ACN) and 50 mM triethyl ammonium bicarbonate (TEAB) (1:1) solution for 30 min. Samples were subsequently alkylated and reduced by adding 25 mM tris (2-carboxyethyl) phosphine hydrochloride (TCEP) and 50 mM TEAB (1:1) and set in thermos mixer at +60 °C with interval mixing for 1 h. After supernatant removal, the samples were submerged with 10× Iodine acetamide in 50 mM TEAB for 30 min in dark. Samples were then washed with 50 mM TEAB:ACN 1:1 solution three times and dried with vacuum concentrator prior to trypsinization (1 μg of trypsin in 50 mM ammonium bicarbonate solution) for 16 h at +37 °C. Obtained peptides were eluted from the gel fragments using 50% ACN, 5% formic acid (FA) solution. Supernatants were again dried using vacuum concentrator, eluted to the analysis buffer (2% Acetonitrile, 0.1% Formic acid), and injected to the NanoLC-MSTOF instrument. All solvents and other materials were purchased from Thermo Fisher Scientific (San Jose, CA, USA) except Trypsin (TPKC treated, Sciex).

### Identification of proteins

Identification of the proteins was done using Protein Pilot® 4.5 (Sciex, Redwood City, USA) and all data dependent analysis (DDA) runs MS/MS spectra were identified against respective Na_v_ channel protein data retrieved from UniprotKB/SwissProt library. FDR 1% and 99% peptide confidence level was used in the library creation and only distinctive peptides were used in the identification. Mass accuracy was set to 5 ppm for each peptide.

### NanoLC-MSTOF parameters

Proteins were analyzed by Nano-RPLC-MSTOF instrumentation using Eksigent 425 NanoLC coupled to high-speed TripleTOF™ 5600+ mass spectrometer (Ab Sciex, Concord, Canada). A microcapillary RP-LC column (cHiPLC® ChromXP C18-CL, 3 μm particle size, 120 Å, 75 μm i.d × 15 cm, Eksigent Concord, Canada) was used for LC separation of peptides. Samples were first loaded into trap column (cHiPLC® ChromXP C18-CL, 3 μm particle size, 120 Å, 75 μm i.d × 5 mm) from autosampler and flushed for 10 min at 2 μl/min (2% ACN, 0.1% FA). The flush system was then switched to line with analytical column. The peptide samples were analyzed with 120 min 6 step gradient using eluent A: 0.1% FA in 1% ACN and eluent B: 0.1% FA in ACN (eluent B from 5 to 7% over 2 min; 7 to 24% over 55 min; 24 to 40% over 29 min; 40 to 60% over 6 min; 60 to 90% over 2 min and kept at 90% for 15 min; 90 to 5% over 0.1 min and kept at 5% for 13 min) at 300 nl/min.

The following key parameters were applied for TripleTOF mass spectrometer in shotgun identification analysis: ion spray voltage floating (ISVF) 2300 V, curtain gas (CUR) 30, interface heater temperature (IHT) +125 °C, ion source gas 1 13, declustering potential (DP) 100 V. Methods were run by Analyst TF 1.5 software (Ab Sciex, USA). For IDA parameters, 0.25 s MS survey scan in the mass range 350–1250 mz was followed by 60 MS/MS scans in the mass range of 100–1500 Da (total cycle time 3.302 s). Switching criteria were set to ions greater than mass to charge ratio (m/z) 350 and smaller than 1250 (m/z) with charge state 2–5 and an abundance threshold of more than 120 counts. Former target ions were excluded for 12 S*. IDA* rolling collision energy (CE) parameters script was used for automatically controlling CE.

### Phagocytosis assay for hESC-derived and mouse RPE

The porcine POS particles were isolated and purified as previously described [75, 78]. Briefly, the eyecups obtained from a slaughterhouse were opened and retinas were removed using forceps under dim red light. The retinas were shaken gently in 0.73 M sucrose phosphate buffer and separated after filtering in sucrose gradient using an ultracentrifuge (Optima ultracentrifuge, Beckman Coulter, Inc., Brea, CA) at 112,400 x g for 1 h at +4 °C. The collected POS layer was centrifuged 3000×*g* for 10 min at +4 °C and stored in 73 mM sucrose phosphate buffer at −80 °C.

The purified POS particles were fed to the hESC-derived RPE cells in a KO-DMEM medium supplemented with 10% fetal bovine serum (FBS) and incubated for either 2 h at RT or 2 h, 4 h or 5 h at +37 °C in 5% CO_2_. In the blocker experiments, selective blockers for Na_v_1.4, Na_v_1.8 and TTX were also added to the medium for the incubation. Then the monolayers were washed twice briefly with PBS and fixed with PFA according to the immunostaining protocol. Phagocytosis was studied in vivo by preparing the mouse eyes under dim red light either at light onset or 2 h and 10 h after it. The mice were reared in normal 12-h light/dark cycle. When blockers were used, the eyecup was opened and then incubated in blocker solutions diluted in Ames’ as described above, for 1 h at +37 °C with the retina left intact.

### Quantification of POS particles in hESC-derived RPE

To detect and quantify POS particles, large random fields were imaged from 3 different samples in each condition with Zeiss LSM780 LSCM (the total number of images in each case is included in the figure legends as “n”). The images were first blurred with a Gaussian function after which a Z-maximum intensity projection was binarized using a global threshold. The number of POS particles was then analyzed from the images converted to mask. In the hESC-derived RPE where subtype Na_v_1.4 had been silenced with lentiviral vectors encoding shRNAs, phagocytosis was analyzed by capturing several fields with a GFP-positive cell in the center of the image. The MIP images were then combined and the average distribution of POS particle labeling was compared between control-GFP (EGFP) construct and the clone TRCN000044419. The image was split into a 3 × 3 grid and the relative intensity of POS labeling was analyzed for each individual square of the grid.

### Statistical analysis of the POS phagocytosis quantification

Each phagocytosis experiment was repeated three times and the images were pooled together. The normality of the data was tested by using Shapiro–Wilk normality test and the differences were first analyzed using ANOVA. Finally, pairwise comparison was conducted by using Kruskal–Wallis test to confirm the possible statistical significance between the experimental conditions.

### Confocal microscopy and image processing

Confocal microscopy was performed with Zeiss LSM780 LSCM on inverted Zeiss Cell Observer microscope (Zeiss, Jena, Germany) by using Plan-Apochromat 63x/1.4 oil immersion objective. Voxel size was set to *x* = *y* = 66 nm and *z* = 200 nm and 1024 × 1024 pixel stacks of 70–120 slices were acquired with line average of 2. The Alexa Fluor 405 was excited with 405 nm diode laser; Alexa Fluor 488 with 488 nm laserline from Argon laser; Alexa Fluor 568 and TRITC with 561 nm DPSS or 562 nm *InTune* laser; Atto 633 and Alexa Fluor 647 with 633 nm HeNe and with 628 nm *InTune* laser. Emission was detected with windows of (in nm) 410–495 (DAPI, Alexa Fluor 405), 499–579 (Alexa Fluor 488), 579–642 (Alexa Fluor 568), and 642–755 (Alexa Fluor 647). Laser powers were minimized to avoid bleaching and photomultiplier tube sensitivities were adjusted to obtain optimal signal-to-noise ratio of the signal. The data was saved in .czi format and deconvolved using Huygens Essential (SVI, Hilversum, Netherlands) software. The deconvolution was performed with theoretical PSF, signal-to-noise ratio of 5 and quality threshold of 0.01. Information regarding the refractive index of the sample was provided by the manufacturer of the ProLong Gold antifade mounting media. Images were further processed with ImageJ [79] and only linear brightness and contrast adjustments were performed for the pixel intensities. Final figures were assembled using Adobe Photoshop CC (2015.5.1 release) and Illustrator CC (2015.3.1 release) (Adobe Systems, San Jose, USA).

## Additional files

Additional file 1: **Figure S1.** Immunolabeling of Na_v_ in hESC-derived and mouse RPE. Z-maximum intensity projections (Z-MIP) of (**a**) hESC-derived and (**b**) mouse RPE stained against Na_v_ channels (green) and tight junction marker ZO-1 (red), together with cross-sectional X-MIPs from the highlighted regions. (PNG 729 kb)

Additional file 2: **Figure S2.** Immunolabeling of different Na_v_ subtypes in hESC-derived and mouse RPE. Different Na_v_ channel subtypes were immunolabeled in (**a**) mature hESC-derived and (**b**) mouse RPE that had been fixed with 1% PFA. Laser scanning confocal microscopy Z-maximum intensity projections of Na_v_ subtypes (green) labeled together with filamentous actin (phalloidin, red). In both samples, the subtypes Na_v_1.1, Na_v_1.3, Na_v_1.5, Na_v_1.7 and Na_v_1.9 showed labeling in cell-cell junctions and apical membrane. The subtype Na_v_1.2 gave extremely weak signals in both samples. Scale bars 10 μm. (PNG 4721 kb)

Additional file 3: **Figure S3.** Immunolabeling of different Na_v_ subtypes during development of hESC-derived RPE. hESC-derived RPE cells were seeded on cell culture inserts and fixed at various timepoints during development. Laser scanning confocal microscopy Z-maximum intensity projections showed that during maturation from 1 d to 9 d after cell seeding, the cellular distribution of subtype Na_v_1.1 stayed homogenous. Contrarily, cellular distribution of subtypes Na_v_1.4 and Na_v_1.5 changed from homogeneous (1 d) to more organized beads (9 d) at the cell-cell junctions (Na_v_1.4) or to bright spots in the cell (Na_v_1.5). The cellular distribution of Na_v_1.8 was initially homogenous but at 9 d, the subtype also showed localization to one or few bright spots in the cells. Scale bars 10 μm. (PNG 1453 kb)

Additional file 4: **Figure S4.** Western blot analysis of different subtypes in hESC-derived RPE. Whole cell lysates of hESC-derived RPE cells were analyzed by electroblotting and the resulting nitrocellulose membranes were stained against the subunits Na_v_1.4-Na_v_1.6 and Na_v_1.8. All subunits showed positive bands between 130 and 250 kDa. The Western blots were used as guides for the gel excision for mass spectrometry analysis. (PNG 83 kb)

Additional file 5: **Figure S5.** Western blot analysis of shRNA knock-down of Na_V_1.4 in ARPE-19 cells. Whole cell lysates of ARPE-19 cells transduced with shRNA expressing EGFP or the lentivirus constructs were analyzed by Western blot. The nitrocellulose membranes were stained against the subunit Na_v_1.4. The staining showed positive bands between 130 and 250 kDa for lysates obtained from EGFP expressing cells as well as cells transduced with shRNA clone 1 (TRCN0000416043) but the labeling intensity was decreased for lysates obtained from cells transduced with the clone 2 (TRCN0000425151) and especially with clone 3 (TRCN0000044419). The labeling band intensity was compared against the β-actin band (between 35 and 55 kDa) that was used as the loading control. Based on the Western blot, the expression for Na_v_1.4 was normalized for EGFP and all shRNA constructs, and we therefore selected clone 3 (TRCN0000044419) for further experiments (Individual datapoints available in Additional file 9: Table S4). (PNG 328 kb)

Additional file 6: **Table S1.** List of chemical and antibody details. (DOCX 46 kb)

Additional file 7: **Table S2.** Individual datapoints for Fig. 1h. (DOCX 55 kb)

Additional file 8: **Table S3.** Individual datapoints for Fig. 6b-d. (DOCX 69 kb)

Additional file 9: **Table S4.** Individual datapoints for Figure S5. (DOCX 37 kb)

## Data Availability

All data generated or analyzed during this study are included in this published article and its supplementary information files. Patch clamp, confocal imaging, and mass spectrometry datasets are available in the Zenodo repository [80]. Where *n* < 6, the individual data values are provided in additional files and cited in the figure legends (Additional file 7: Table S2, Additional file 8: Table S3, Additional file 9: Table S4).
