# Supplementary figures and images for: Sodium channels enable fast electrical signaling and regulate phagocytosis in the retinal pigment epithelium
Source: BMC Biol. 2019 Aug 15;17:63. doi: 10.1186/s12915-019-0681-1 (PMC6694495; doi:10.1186/s12915-019-0681-1)

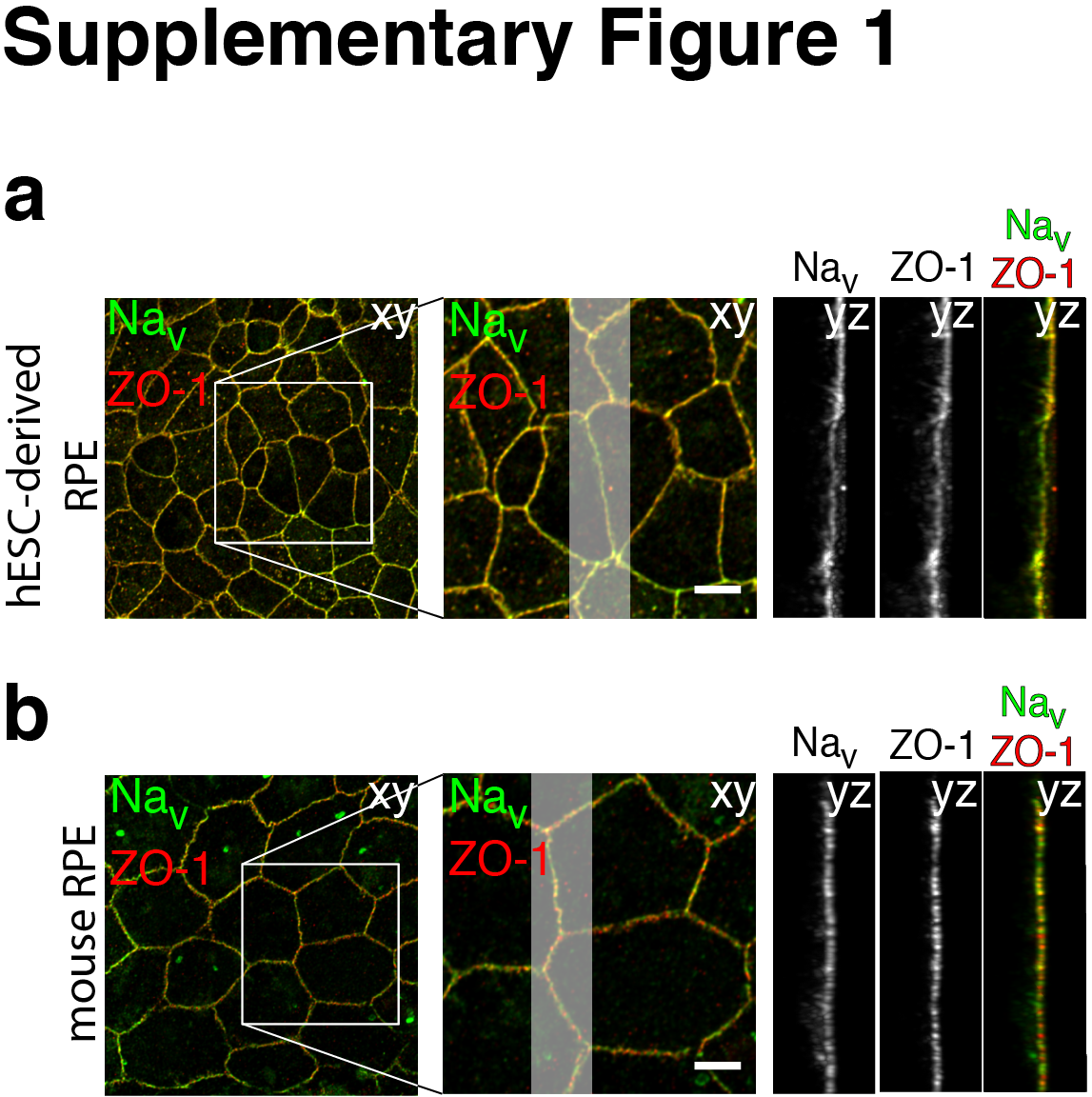

Supplement: Supplementary file 1 — Figure S1. Immunolabeling of Nav in hESC-derived and mouse RPE. Z-maximum intensity projections (Z-MIP) of (a) hESC-derived and (b) mouse RPE stained against Nav channels (green) and tight junction marker ZO-1 (red), together with cross-sectional X-MIPs from the highlighted regions. (PNG 729 kb) [file 12915_2019_681_MOESM1_ESM.png]

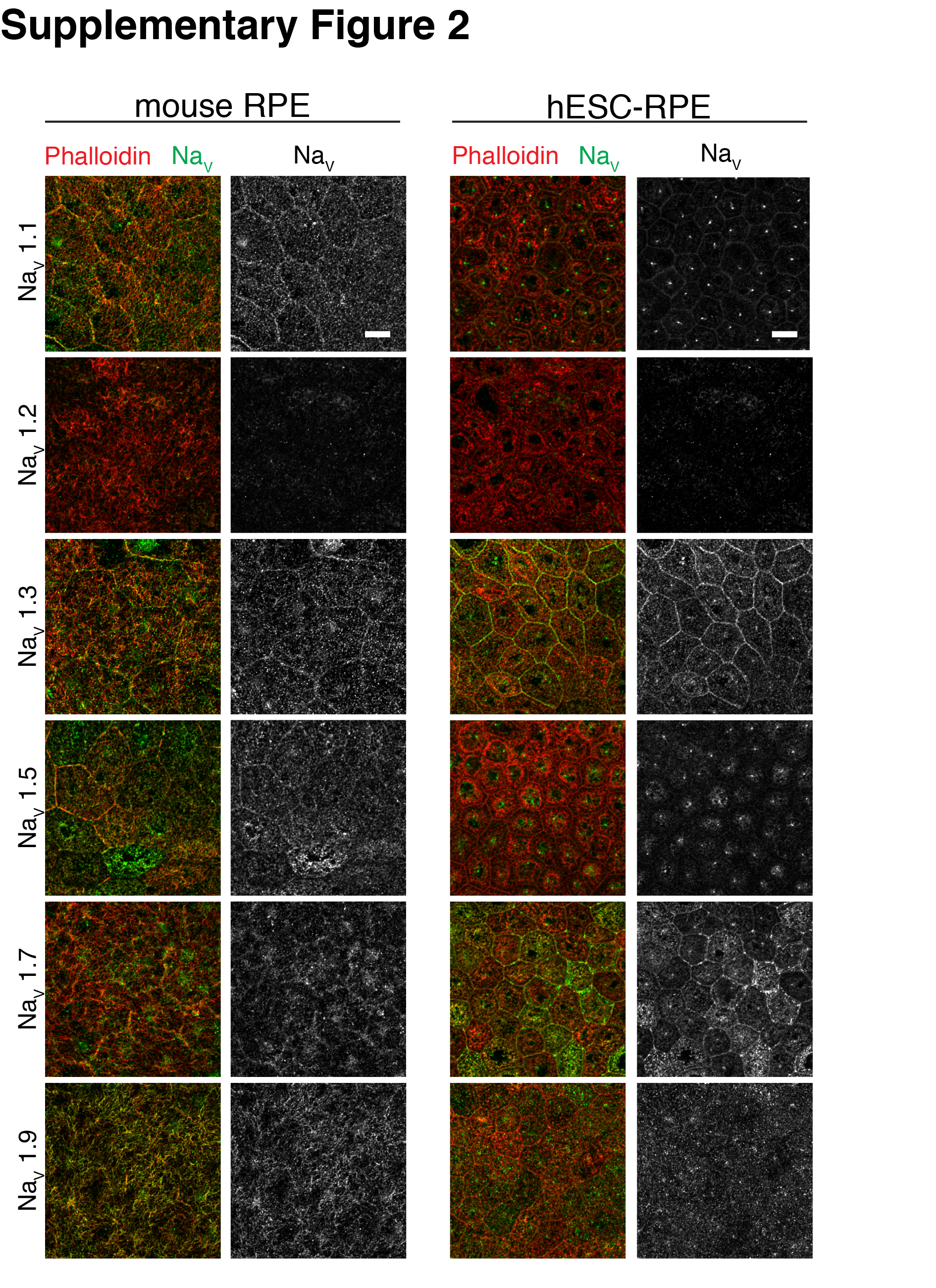

Supplement: Supplementary file 2 — Figure S2. Immunolabeling of different Nav subtypes in hESC-derived and mouse RPE. Different Nav channel subtypes were immunolabeled in (a) mature hESC-derived and (b) mouse RPE that had been fixed with 1% PFA. Laser scanning confocal microscopy Z-maximum intensity projections of Nav subtypes (green) labeled together with filamentous actin (phalloidin, red). In both samples, the subtypes Nav1.1, Nav1.3, Nav1.5, Nav1.7 and Nav1.9 showed labeling in cell-cell junctions and apical membrane. The subtype Nav1.2 gave extremely weak signals in both samples. Scale bars 10 μm. (PNG 4721 kb) [file 12915_2019_681_MOESM2_ESM.png]

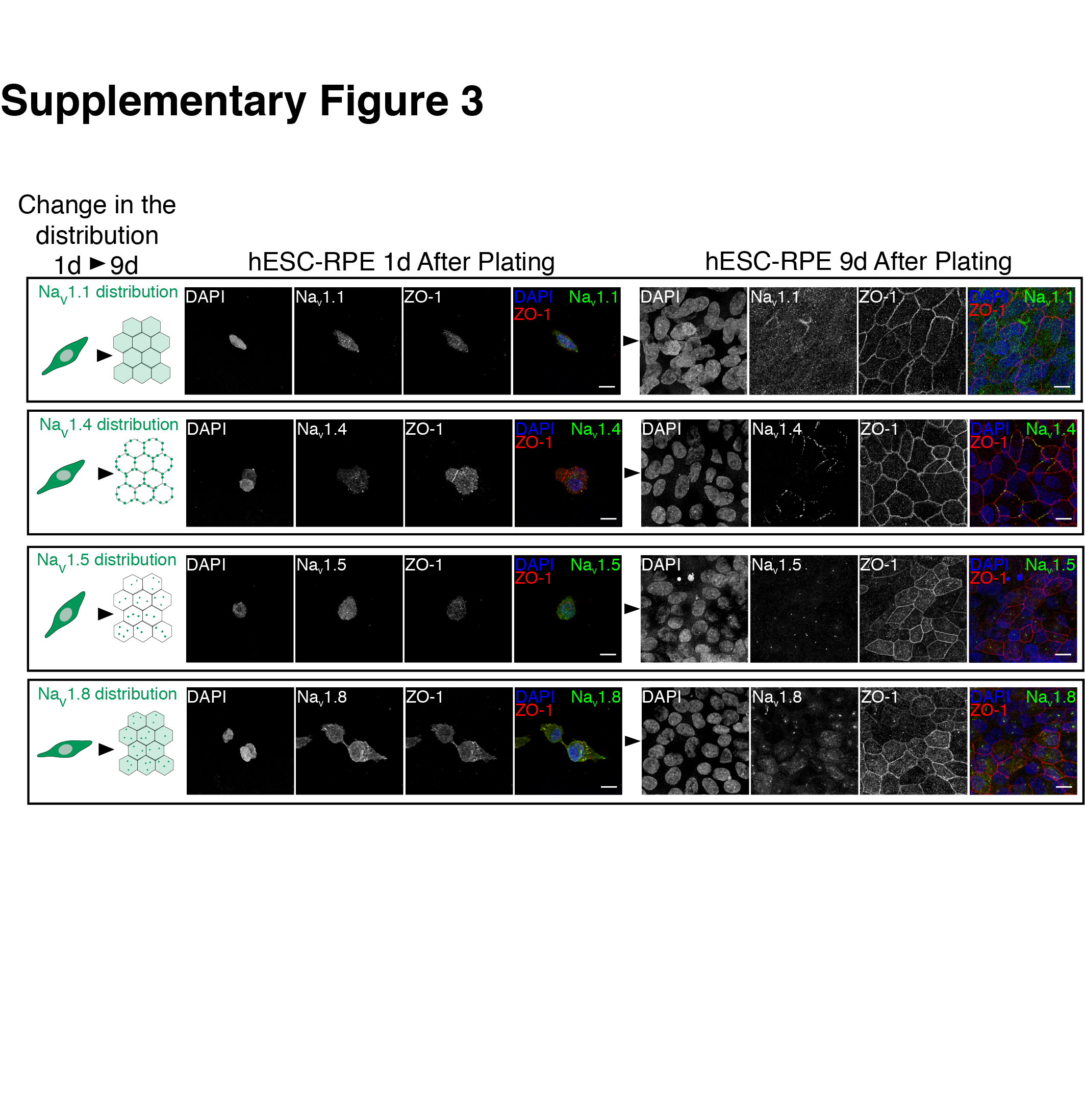

Supplement: Supplementary file 3 — Figure S3. Immunolabeling of different Nav subtypes during development of hESC-derived RPE. hESC-derived RPE cells were seeded on cell culture inserts and fixed at various timepoints during development. Laser scanning confocal microscopy Z-maximum intensity projections showed that during maturation from 1 d to 9 d after cell seeding, the cellular distribution of subtype Nav1.1 stayed homogenous. Contrarily, cellular distribution of subtypes Nav1.4 and Nav1.5 changed from homogeneous (1 d) to more organized beads (9 d) at the cell-cell junctions (Nav1.4) or to bright spots in the cell (Nav1.5). The cellular distribution of Nav1.8 was initially homogenous but at 9 d, the subtype also showed localization to one or few bright spots in the cells. Scale bars 10 μm. (PNG 1453 kb) [file 12915_2019_681_MOESM3_ESM.png]

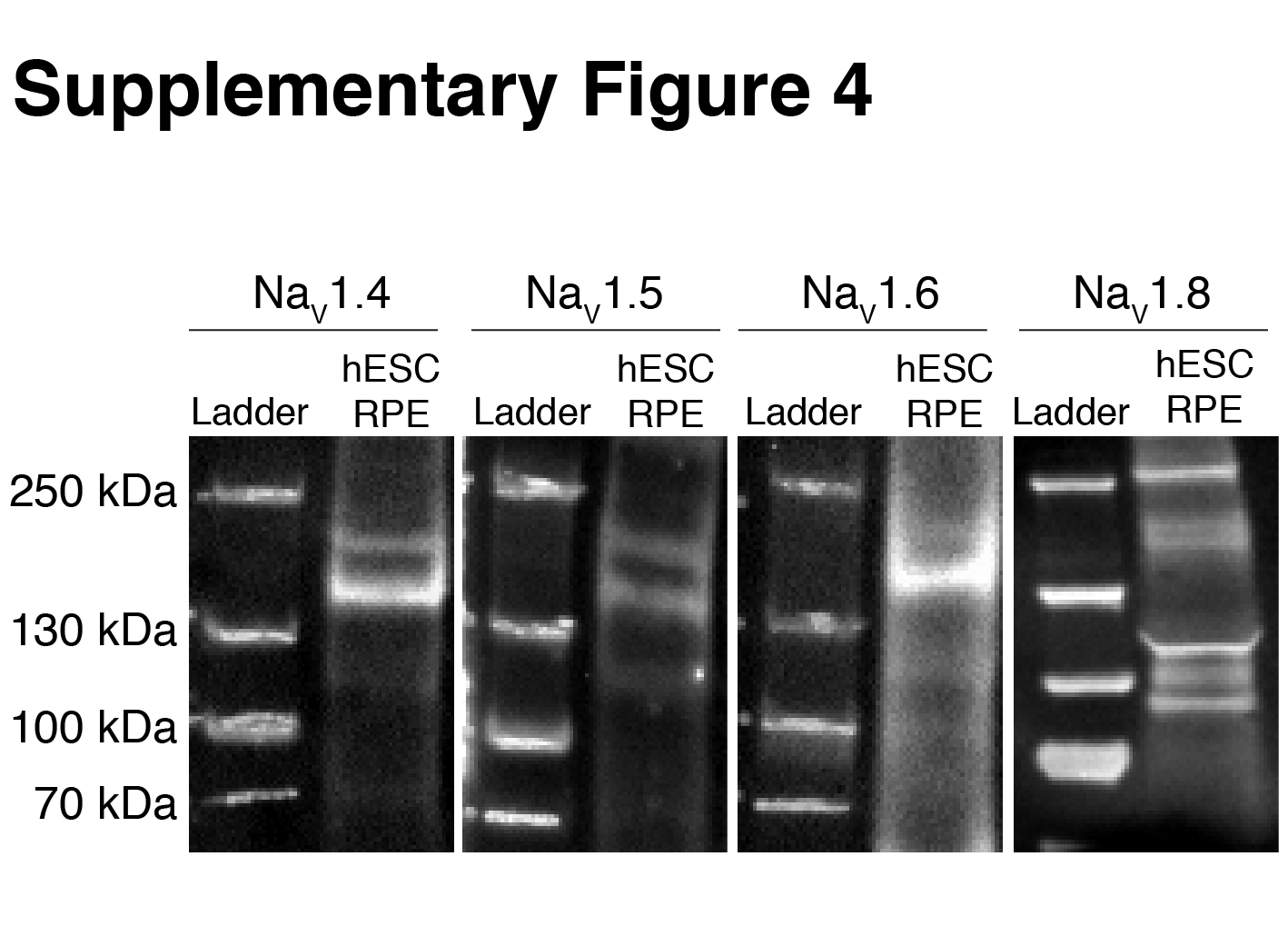

Supplement: Supplementary file 4 — Figure S4. Western blot analysis of different subtypes in hESC-derived RPE. Whole cell lysates of hESC-derived RPE cells were analyzed by electroblotting and the resulting nitrocellulose membranes were stained against the subunits Nav1.4-Nav1.6 and Nav1.8. All subunits showed positive bands between 130 and 250 kDa. The Western blots were used as guides for the gel excision for mass spectrometry analysis. (PNG 83 kb) [file 12915_2019_681_MOESM4_ESM.png]

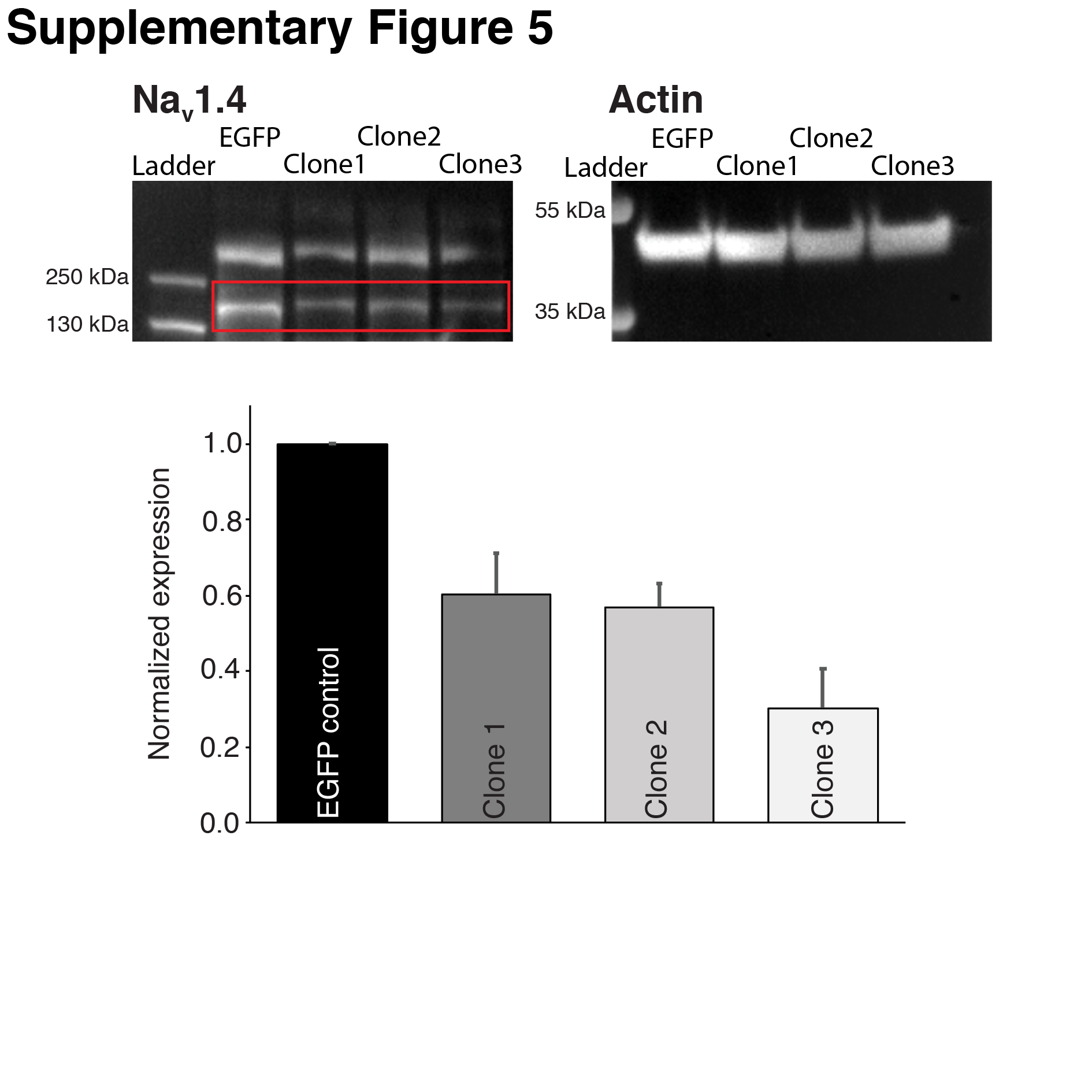

Supplement: Supplementary file 5 — Figure S5. Western blot analysis of shRNA knock-down of NaV1.4 in ARPE-19 cells. Whole cell lysates of ARPE-19 cells transduced with shRNA expressing EGFP or the lentivirus constructs were analyzed by Western blot. The nitrocellulose membranes were stained against the subunit Nav1.4. The staining showed positive bands between 130 and 250 kDa for lysates obtained from EGFP expressing cells as well as cells transduced with shRNA clone 1 (TRCN0000416043) but the labeling intensity was decreased for lysates obtained from cells transduced with the clone 2 (TRCN0000425151) and especially with clone 3 (TRCN0000044419). The labeling band intensity was compared against the β-actin band (between 35 and 55 kDa) that was used as the loading control. Based on the Western blot, the expression for Nav1.4 was normalized for EGFP and all shRNA constructs, and we therefore selected clone 3 (TRCN0000044419) for further experiments (Individual datapoints available in Additional file 9: Table S4). (PNG 328 kb) [file 12915_2019_681_MOESM5_ESM.png]
