# Supplementary material for: Sodium channels enable fast electrical signaling and regulate phagocytosis in the retinal pigment epithelium
Source: BMC Biol. 2019 Aug 15;17:63. doi: 10.1186/s12915-019-0681-1 (PMC6694495; doi:10.1186/s12915-019-0681-1)
Supplement: Supplementary file 6 — Table S1. List of chemical and antibody details. (DOCX 46 kb) [file 12915_2019_681_MOESM6_ESM.docx]

| Antibody | Company | Catalog Number | RRID | LOT |
| --- | --- | --- | --- | --- |
| CRALBP | Abcam | ab15051 | AB_2269474 | GR229880-2 |
| Na_v_1.1 | Alomone Labs | ASC-001 | AB_2040003 | ASC001AN2225 |
| Na_v_1.2 | Abcam | ab99044 | AB_10675393 |  |
| Na_v_1.3 | Alomone Labs | ASC-004 | AB_2040007 | ASC004AN150 |
| Na_v_1.4 | Alomone Labs | ASC-020 | AB_2040009 | ASC020AN0150 |
| SCN4A | Thermo Fisher Scientific | PA5-36989 | AB_2553851 | SL2450041L |
| Na_v_1.5 | Alomone Labs | AGP-008 | AB_2340960 | ASC005AN3350 |
| Na_v_1.6 | Alomone Labs | ASC-009 | AB_2040202 | ASC009AN2550 |
| Na_v_1.7 | Alomone Labs | ASC-008 | AB_2040198 | ASC008AN1125 |
| Na_v_1.8 | Alomone labs | AGP-029 | AB_2340965 | AGP029AN0102 |
| Na_v_1.9 | Alomone labs | AGP-030 | AB_2340966 | AGP030AN0150 |
| Pan Na_v_ | Alomone labs | ASC-003 | AB_2040204 | ASC003AN3102 |
| Zonula occludens-1 (ZO-1) | Thermo Fisher Scientific | 33-9100 | AB_2533147 | AA260617 |
| Anti-beta Actin antibody [AC-15] | Abcam | ab6276 | AB_2223210 | GR3182323-2 |
| Goat anti-Rabbit IgG (H+L) Alexa Fluor 568 | Thermo Fisher Scientific | A-11011 | AB_143157 | 1892091 |
| Donkey anti-rabbit IgG (H+L) Alexa Fluor 488 | Thermo Fisher Scientific | A-11008 | AB_143165 | 1674651 |
| Donkey anti-mouse IgG (H+L) Alexa Fluor 568 | Thermo Fisher Scientific | A10037 | AB_2534013 | 1995837 |
| Donkey anti-mouse IgG (H+L) Alexa Fluor 488 | Thermo Fisher Scientific | A-21202 | AB_141607 | 1915874 |
| Goat Anti-Guinea Pig IgG (H+L) Alexa Fluor 568 | Thermo Fisher Scientific | A-11075 | AB_2534119 | 1642965 |
| Goat anti-Mouse IgG (H+L) Alexa Fluor 488 | Thermo Fisher Scientific | A-11029 | AB_2534069 | 1911842 |
| Donkey Anti-Rabbit IgG (H+L) Alexa Fluor 647 | Thermo Fisher Scientific | A-31573 | AB_2536183 | 1322326 |
| Goat anti-Mouse IgG (H+L) Alexa Fluor 647 | Thermo Fisher Scientific | A-21236 | AB_2535805 | 1793803 |
| Goat Anti-Guinea Pig IgG (H+L) Alexa Fluor 647 | Thermo Fisher Scientific | A-21450 | AB_2735091 | 1711474 |
| Goat anti-Mouse IgG (H+L) Alexa Fluor 405 | Thermo Fisher Scientific | A-31553 | AB_221604 | 1711490 |
| Alexa Fluor® 647 Phalloidin antibody | Thermo Fisher Scientific | A22287 | AB_2620155 | 1948974 |
| Phalloidin–Atto 633 | Sigma-Aldrich | 68825 | n/a | BCBZ7901 |
| Phalloidin-TRITC | Sigma-Aldrich | P1951 | AB_2315148 | SLBM2123V |
| Goat Anti-Rabbit IgG - H&L Polyclonal antibody, Hrp Conjugated | Abcam | ab6721 | AB_955447 | GR3182323-2 |
| Goat Anti-Guinea pig IgG - H&L Polyclonal antibody, Hrp Conjugated | Abcam | ab6908 | AB_955425 | GR3177574-6 |
| HRP Horse Anti-Mouse IgG Antibody | Vector Laboratories | PI-2000 | AB_2336177 | X0328 |
| Nanogold – Fab goat anti rabbit IgG (H+L) | Nanoprobes | 2004 | AB_2802149 | 33C827 |
| Nanogold – Fab goat anti guinea pig IgG | Nanoprobes | 2055 | AB_2802149 | 30C496 |
| Nanogold – Fab goat anti mouse IgG (H+L) | Nanoprobes | 2002 | AB_2637031 | 33C537 |

Other reagents

| Ames’ Medium | Sigma-Aldrich | A1420-10X1L | SLBH3527V |
| --- | --- | --- | --- |
| Poly-L-lysine solution | Sigma-Aldrich | P4832-50ML | RNBG7320 |
| TrypLE™ Select Enzyme (1X), no phenol red | Thermo Fisher Scientific | 12563011 | 02502113 |
| Menzel Gläser | VWR | 630-1985 | 7970578 |
| Collagen from human placenta | Sigma-Aldrich | C5533 | 055M4001 |
| Costar® 24-well Clear Not Treated Multiple Well Plates, Bulk Pack, Sterile | Costar | 3738 | 18018033s |
| Hanging cell culture insert | Millipore | PIRP12R48 | 15060116 |
| KO-DMEM | Gibco | 10829-018 | 1666834 |
| KO-SR | Gibco | 10828-028 | 1667387 |
| GlutaMAX | Gibco | 35050-038 | 1717649 |
| NEAA | Lonza | BE13-1ME | 4MB113 |
| Beta-mercaptoethanol | Gibco | 31350-010 | 1721714 |
| DMEM/F12 | Gibco | 31331028 | 1976787 |
| Trypsin-EDTA | Thermo Fisher Scientific | 25200-056 | 2010314 |
| DPBS, 10x, no calcium, no magnesium | Gibco | 14200-067 | 1976769 |
| 4,9-Anhydrotetrodotoxin | Focus Biomolecules | 10-3700 | FBA1026b |
| µ-Conotoxin GIIB | Alomone Labs | C-270CN311 | C270CN2858 |
| Tetrodotoxin | Tocris | ASC-055 | 44B |
| A-803467 | Sigma-Aldrich | A3109 | 043M4727V |
| DMSO | Sigma-Aldrich | D4540 | RNBC923 |
| Paraformaldehyde 20% Solution, EM grade | VWR | 15713-S | 180605-11 |
| TBS, Tris Buffered Saline, 10X Solution, pH 7.4 | Thermo Fisher Scientific | BP2471-1 | 186187 |
| ProLong Gold antifade mounting medium | Thermo Fisher Scientific | P36935 | 19907459 |
| APEX labeling kit | Thermo Fisher Scientific | A10468 | 1620131 |
| Bovine Serum Albumin | Sigma-Aldrich | A8022 | SLBW8359 |
| Triton™ X-100 | Sigma-Aldrich | T8787 | SLBV4122 |
| TWEEN® 20 | Sigma-Aldrich | P4916 | SLBR6201V |
| Cover glasses, high performance, D=0.17mm, box with 1000 pc. | Zeiss | 474030-9000-000 | 38374017 |
| 20X Bolt™ MES SDS Running Buffer | Thermo Fisher Scientific | B0002 | 1991197 |
| NuPAGE™ LDS Sample Buffer (4X) | Thermo Fisher Scientific | NP0007 | 1962150 |
| NuPAGE™ 3-8% Tris-Acetate Protein Gels, 1.0 mm, 10-well | Thermo Fisher Scientific | EA0375BOX | 17101071 |
| Bolt™ 4-12% Bis-Tris Plus Gels, 10-well | Thermo Fisher Scientific | NW04120BOX | 19042370 |
| PageRuler™ Plus Prestained Protein Ladder, 10 to 250 kDa | Thermo Fisher Scientific | 26619 | 00750250 |
| Trans-Blot® Turbo™ RTA Mini Nitrocellulose Transfer Kit, for 40 blots | Bio-Rad | 1704270 | L002043A  L002049A  L002051E |
| Western Bright ECL Kit | AH Diagnostics | K-12045-D20 | 180713-15 |
| HEPES | Sigma-Aldrich | H3375-1KG | SLBV6923 |
| KCl | Sigma-Aldrich | P9541 | BCBQ0895V |
| NaCl | Sigma-Aldrich | S9888-1KG | SLBR9752V |
| TeaCl | Sigma-Aldrich | T2265 | BCBQ7343 |
| CsMSO | Sigma-Aldrich | C1426-5G | MKBT4630V |
| K-gluconate | Sigma-Aldrich | G4500 | 060M02462V |
| CsCl | Sigma-Aldrich | 289329-25G | MKBP3568V |
| EGTA | Sigma-Aldrich | E3889-25G | 1002268742 |
| CaCl_2_ | Sigma-Aldrich | C1016 | BCBG5219V |
| Mg-ATP | Sigma-Aldrich | A9187-1G | SLBR2781V |
| Na-GTP | Sigma-Aldrich | G8877-1G | SLBZ2392 |
| Penicillin-Streptomycin (10,000 U/mL) | Thermo Fisher Scientific | 15140122 | 2041563 |
| Sodium acetate | Sigma-Aldrich | S2889-250G | SLBV8347 |
| Saponin | Sigma-Aldrich | 47036-50G-F | BCBL7176V |
| Ammonium chloride | AnalytiCal | 419417 | 1687M100 |
| HQ Silver Enhancement Kit | Nanoprobes | 2019 | 33C597 |
| Glutaraldehyde | Electron microscopy sciences | 16120 | 1120717 |
| Sodium dihydrogen phosphate monohydrate | Merck | A689746 634 | 1.06346.1000 |
| di-Sodium hydrogen phosphate dihydrate | Merck | K36424880 641 | 1.06580.1000 |
| Paraformaldehyde | Sigma-Aldrich | P6148-1KG | SZBG1020V |
| L-Lysine monohydrochloride | Sigma-Aldrich | L5426-100G | SLBM5436V |
| BSA | HyClone | SH30574.02 | AB10187986A |
| Glycine | Sigma-Aldrich | 33226-1KG | SZBF1660V |
| Sodium thiosulfate pentra-hydrate | Sigma-Aldrich | 217247-500G | SZBC3450V |
| Sodium meta-Periodate | Pierce | 20504 | GE96842 |
| Gold(III) chloride trihydrate | Sigma-Aldrich | 520918 | MKCF1650 |
| Sodium hydroxide | Merck | HC60130513 | 1.09913.0001 |
| Cesium hydroxide | Sigma-Aldrich | 232068-100G | SHBF8933V |
| PageBlue Protein Staining Solution | Thermo Fisher Scientific | 24620 | 0041775 |
| Acetonitrile | WVR | 20060.320 | 13L023917 |
| Formic acid | Honeywell Research Chemicals | 56302_50ML | H1630 |
| Triethylammonium bicarbonate buffer | Honeywell Research Chemicals | 17902-100ML | H337CIL |
| Iodoacetamide | Sigma-Aldrich | I6125-5G | SLBP7486V |
