# Supplementary material for: Sodium channels enable fast electrical signaling and regulate phagocytosis in the retinal pigment epithelium
Source: BMC Biol. 2019 Aug 15;17:63. doi: 10.1186/s12915-019-0681-1 (PMC6694495; doi:10.1186/s12915-019-0681-1)
Supplement: Supplementary file 7 — Table S2. Individual datapoints for Fig. 1h. (DOCX 55 kb) [file 12915_2019_681_MOESM7_ESM.docx]

Fig 1 h

| **τ_recovery_** | cell 1 | | cell 2 | | cell 3 | | cell 4 | |
| --- | --- | --- | --- | --- | --- | --- | --- | --- |
|  | Response amplitude | Normalized response | Response amplitude | Normalized response | Response amplitude | Normalized response | Response amplitude | Normalized response |
| Time (ms) | (pA) |  | (pA) |  | (pA) |  | (pA) |  |
| Test pulse | -84.5 | 1 | -73.4 | 1 | -285.6 | 1 | -126.5 | 1 |
| 10 | -22.9 | 0.271 | -14.5 | 0.198 | -55.7 | 0.195 | -10.4 | 0.082 |
| 30 | -42 | 0.497 | -26 | 0.354 | -157 | 0.550 | -48.2 | 0.381 |
| 50 | -67.4 | 0.798 | -49.8 | 0.678 | -196.2 | 0.687 | -64.7 | 0.511 |
| 70 | -60 | 0.710 | -59.5 | 0.810 | -257.3 | 0.901 | -103 | 0.814 |
| 90 | -79.3 | 0.938 | -70 | 0.953 | -247 | 0.865 | -104.2 | 0.824 |
| 110 | -70.3 | 0.832 | -74.5 | 1.015 | -280.8 | 0.983 | -104.7 | 0.828 |
| 130 | -82 | 0.970 | -74.6 | 1.016 | -260 | 0.910 | -113.8 | 0.900 |
| 150 | -84 | 0.994 | -82.6 | 1.125 | -294.6 | 1.0312 | -126.6 | 1.001 |
| 170 | -88.5 | 1.047 | -76.3 | 1.039 | -277.4 | 0.971 | -133 | 1.051 |
| 190 | -87.1 | 1.031 | -78.6 | 1.071 | -319.2 | 1.118 | -134.3 | 1.062 |
| 210 | -83.5 | 0.988 | -81 | 1.104 | -310.4 | 1.087 | -121.8 | 0.963 |
| 230 | -86.7 | 1.026 | -82.7 | 1.127 | -306.2 | 1.072 | -139.3 | 1.101 |
| 250 | -92.8 | 1.098 | -88.8 | 1.210 | -306.2 | 1.072 | -137.3 | 1.085 |
| 270 | -81.5 | 0.964 | -83 | 1.131 | -320.1 | 1.121 | -132.1 | 1.044 |

| **τ_recovery_** | cell 5 | |
| --- | --- | --- |
|  | Response amplitude | Normalized response |
| Time (ms) | (pA) |  |
| Test pulse | -108.2 | 1 |
| 10 | -10.1 | 0.093 |
| 30 | -38.3 | 0.354 |
| 50 | -68 | 0.628 |
| 70 | -87.2 | 0.806 |
| 90 | -91.5 | 0.846 |
| 110 | -97.7 | 0.903 |
| 130 | -118.4 | 1.094 |
| 150 | -113.2 | 1.046 |
| 170 | -117.3 | 1.084 |
| 190 | -118.2 | 1.092 |
| 210 | -117.3 | 1.084 |
| 230 | -120.4 | 1.113 |
| 250 | -140.4 | 1.298 |
| 270 | -120 | 1.109 |
