# Supplementary material for: Sodium channels enable fast electrical signaling and regulate phagocytosis in the retinal pigment epithelium
Source: BMC Biol. 2019 Aug 15;17:63. doi: 10.1186/s12915-019-0681-1 (PMC6694495; doi:10.1186/s12915-019-0681-1)
Supplement: Supplementary file 8 — Table S3. Individual datapoints for Fig. 6b-d. (DOCX 69 kb) [file 12915_2019_681_MOESM8_ESM.docx]

Fig 6 b-d

| **Control** | cell 1 | | cell 2 | | cell 3 | | cell 4 | |
| --- | --- | --- | --- | --- | --- | --- | --- | --- |
|  | Control | Conotoxin | Control | Conotoxin | Control | Conotoxin | Control | Conotoxin |
| Voltage (mV) | Current  (pA) | Current (pA) | Current (pA) | Current  (pA) | Current (pA) | Current (pA) | Current (pA) | Current (pA) |
| -80 | -4.5 | 0.3 | -3.1 | -1.8 | -1.0 | -1.2 | 4.9 | 3.5 |
| -70 | -8.7 | -5.3 | -8.0 | -6.6 | -6.6 | -7.7 | -3.8 | -3.6 |
| -60 | -13.7 | -8.2 | -6.7 | -12.1 | -11.4 | -6.0 | -7.2 | -6.8 |
| -50 | -15.0 | -11.9 | -8.0 | -14.5 | -11.5 | -12.3 | -14.6 | -14.6 |
| -40 | -33.7 | -29.6 | -26.0 | -32.7 | -41.2 | -42.4 | -41.3 | -52.6 |
| -30 | -97.6 | -71.9 | -58.1 | -61.4 | -100.6 | -106.2 | -108.7 | -112.1 |
| -20 | -167.6 | -120.7 | -100.1 | -100.2 | -145.9 | -126.3 | -153.5 | -136.2 |
| -10 | -190.8 | -139.2 | -118.8 | -115.4 | -170.0 | -143.1 | -158.6 | -143.0 |
| 0 | -185.6 | -135.3 | -125.7 | -110.3 | -158.4 | -139.7 | -162.9 | -146.7 |
| 10 | -181.7 | -134.4 | -129.5 | -109.6 | -152.4 | -125.7 | -148.1 | -140.2 |
| 20 | -163.9 | -122.4 | -109.6 | -93.6 | -136.3 | -114.4 | -145.9 | -119.6 |
| 30 | -151.2 | -115.5 | -103.3 | -91.6 | -121.1 | -101.1 | -127.7 | -114.3 |
| 40 | -131.0 | -103.5 | -95.7 | -72.8 | -109.6 | -88.2 | -112.8 | -105.2 |
| 50 | -113.1 | -97.7 | -78.4 | -63.9 | -93.5 | -77.1 | -100.7 | -89.0 |
| 60 | -108.2 | -91.7 | -67.7 | -51.9 | -87.2 | -69.5 | -93.9 | -80.2 |

| **EGFP** | cell 1 | | cell 2 | | cell 3 | |
| --- | --- | --- | --- | --- | --- | --- |
|  | Control | Conotoxin | Control | Conotoxin | Control | Conotoxin |
| Voltage (mV) | Current (pA) | Current (pA) | Current (pA) | Current  (pA) | Current (pA) | Current (pA) |
| -80 | -10.3 | -9.5 | -7.0 | -4.6 | -7.0 | -5.4 |
| -70 | -6.3 | -4.1 | -6.3 | -6.2 | -6.0 | -7.0 |
| -60 | -4.0 | -5.3 | -9.1 | -10.0 | -10.9 | -5.4 |
| -50 | -10.8 | -10.1 | -13.3 | -14.5 | -12.9 | -8.3 |
| -40 | -33.3 | -31.4 | -44.3 | -48.8 | -27.3 | -31.2 |
| -30 | -117.3 | -71.7 | -162.1 | -135.1 | -78.7 | -77.4 |
| -20 | -155.4 | -106.9 | -245.3 | -171.6 | -124.0 | -107.2 |
| -10 | -172.2 | -114.5 | -271.7 | -189.4 | -140.5 | -118.6 |
| 0 | -158.4 | -115.6 | -258.9 | -172.2 | -132.1 | -114.4 |
| 10 | -149.3 | -99.8 | -250.1 | -161.0 | -123.7 | -104.1 |
| 20 | -135.7 | -86.0 | -228.8 | -154.0 | -111.45 | -93.4 |
| 30 | -121.7 | -83.0 | -200.3 | -129.8 | -95.8 | -84.8 |
| 40 | -109.6 | -74.8 | -184.4 | -122.4 | -79.8 | -71.1 |
| 50 | -99.1 | -70.5 | -156.3 | -104.0 | -64.7 | -65.6 |
| 60 | -84.5 | -54.4 | -134.9 | -95.8 | -52.7 | -54.8 |

| **clone 419** | cell 1 | | cell 2 | | cell 3 | |
| --- | --- | --- | --- | --- | --- | --- |
|  | Control | Conotoxin | Control | Conotoxin | Control | Conotoxin |
| Voltage (mV) | Current (pA) | Current (pA) | Current (pA) | Current  (pA) | Current (pA) | Current (pA) |
| -80 | 0.5 | -1.2 | 1.0 | -0.8 | -2.0 | -4.5 |
| -70 | -4.6 | -5.1 | -3.0 | -4.9 | -5.2 | -9.0 |
| -60 | -4.3 | -7.2 | -6.5 | -6.0 | -8.4 | -14.0 |
| -50 | -8.7 | -10.3 | -20.3 | -9.1 | -11.8 | -12.3 |
| -40 | -18.8 | -28.0 | -14.1 | -13.8 | -13.5 | -14.6 |
| -30 | -52.7 | -76.1 | -22.8 | -23.1 | -22.7 | -20.5 |
| -20 | -98.7 | -116.5 | -35.1 | -30.6 | -31.2 | -27.6 |
| -10 | -118.7 | -133.4 | -41.8 | -35.6 | -45.2 | -38.9 |
| 0 | -127.8 | -133.0 | -42.4 | -38.1 | -45.7 | -42.3 |
| 10 | -118.4 | -121.9 | -43.7 | -35.6 | -45.0 | -42.6 |
| 20 | -106.9 | -110.7 | -50.8 | -34.1 | -46.2 | -44.6 |
| 30 | -106.3 | -98.3 | -46.2 | -44.4 | -54.1 | -40.6 |
| 40 | -88.7 | -93.8 | -50.2 | -38.2 | -62.8 | -38.6 |
| 50 | -83.6 | -80.5 | -49.4 | -35.3 | -43.7 | -43.1 |
| 60 | -78.4 | -77.2 | -49.3 | -29.0 | -58.8 | -45.9 |
