# Supplementary material for: Sodium channels enable fast electrical signaling and regulate phagocytosis in the retinal pigment epithelium
Source: BMC Biol. 2019 Aug 15;17:63. doi: 10.1186/s12915-019-0681-1 (PMC6694495; doi:10.1186/s12915-019-0681-1)
Supplement: Supplementary file 9 — Table S4. Individual datapoints for Figure S5. (DOCX 37 kb) [file 12915_2019_681_MOESM9_ESM.docx]

Fig S5

**Gel 1**

| **Na_v_ blot** |  |  |  |  |
| --- | --- | --- | --- | --- |
| **Gel background** | 24.3 |  |  |  |
|  | **Control** | **Clone1** | **Clone2** | **Clone3** |
| **Band intensity** | 91.2 | 48.8 | 52.1 | 38.0 |
| **Background subtracted** | 66.9 | 24.5 | 27.8 | 13.7 |
| **Na_v_ normalized to max intensity** | 1 | 0.37 | 0.42 | 0.20 |
|  |  |  |  |  |
| **Actin blot** |  |  |  |  |
| **Gel background** | 7.4 |  |  |  |
|  | **Control** | **Clone1** | **Clone2** | **Clone3** |
| **Band intensity** | 199.2 | 152.3 | 132.9 | 170.3 |
| **Background subtracted** | 191.8 | 144.9 | 125.5 | 162.9 |
| **Actin normalized to max intensity** | 1 | 0.76 | 0.65 | 0.85 |
|  |  |  |  |  |
| **Norm. Na_v_ / Norm. Actin** |  |  |  |  |
|  | **Control** | **Clone1** | **Clone2** | **Clone3** |
|  | **1** | **0.49** | **0.64** | **0.24** |

**Gel 2**

| **Na_v_ blot** |  |  |  |  |
| --- | --- | --- | --- | --- |
| **Gel background** | 17.2 |  |  |  |
|  | **Control** | **Clone1** | **Clone2** | **Clone3** |
| **Band intensity** | 77.8 | 52.8 | 37.6 | 27.8 |
| **Background subtracted** | 60.6 | 35.6 | 20.4 | 10.6 |
| **Na_v_ normalized to max intensity** | 1 | 0.59 | 0.34 | 0.17 |
|  |  |  |  |  |
| **Actin blot** |  |  |  |  |
| **Gel background** | 8.3 |  |  |  |
|  | **Control** | **Clone1** | **Clone2** | **Clone3** |
| **Band intensity** | 223.3 | 208.7 | 150.6 | 162.3 |
| **Background subtracted** | 215.0 | 200.4 | 142.3 | 154.0 |
| **Actin normalized to max intensity** | 1 | 0.93 | 0.66 | 0.72 |
|  |  |  |  |  |
| **Norm. Na_v_ / Norm. Actin** |  |  |  |  |
|  | **Control** | **Clone1** | **Clone2** | **Clone3** |
|  | **1** | **0.63** | **0.51** | **0.24** |

**Gel 3**

| **Na_v_ blot** |  |  |  |  |
| --- | --- | --- | --- | --- |
| **Gel background** | 17.2 |  |  |  |
|  | **Control** | **Clone1** | **Clone2** | **Clone3** |
| **Band intensity** | 98.4 | 57.3 | 44.4 | 37.3 |
| **Background subtracted** | 79.9 | 38.8 | 25.9 | 18.8 |
| **Na_v_ normalized to max intensity** | 1 | 0.49 | 0.32 | 0.23 |
|  |  |  |  |  |
| **Actin blot** |  |  |  |  |
| **Gel background** | 8.3 |  |  |  |
|  | **Control** | **Clone1** | **Clone2** | **Clone3** |
| **Band intensity** | 241.1 | 171.2 | 142.0 | 136.8 |
| **Background subtracted** | 234.3 | 164.4 | 135.2 | 130 |
| **Actin normalized to max intensity** | 1 | 0.70 | 0.58 | 0.55 |
|  |  |  |  |  |
| **Norm. Na_v_ / Norm. Actin** |  |  |  |  |
|  | **Control** | **Clone1** | **Clone2** | **Clone3** |
|  | **1** | **0.69** | **0.56** | **0.42** |

| **Average (gels 1-3)** |  |  |  |  |
| --- | --- | --- | --- | --- |
|  |  |  |  |  |
|  |  |  |  |  |
|  |  |  |  |  |
| **Normalized Na_v_ expression** |  |  |  |  |
|  | **Control** | **Clone1** | **Clone2** | **Clone3** |
| **Average** | 1 | 0.60 | 0.57 | 0.30 |
| **STDEV** | 0 | 0.11 | 0.06 | 0.10 |
